# Neuroinflammatory processes are augmented in mice overexpressing human heat-shock protein B1 following ethanol-induced brain injury

**DOI:** 10.1186/s12974-020-02070-2

**Published:** 2021-01-10

**Authors:** Brigitta Dukay, Fruzsina R. Walter, Judit P. Vigh, Beáta Barabási, Petra Hajdu, Tamás Balassa, Ede Migh, András Kincses, Zsófia Hoyk, Titanilla Szögi, Emőke Borbély, Bálint Csoboz, Péter Horváth, Lívia Fülöp, Botond Penke, László Vígh, Mária A. Deli, Miklós Sántha, Melinda E. Tóth

**Affiliations:** 1 Institute of Biochemistry, Biological Research Centre, Temesvári krt. 62, Szeged, H-6726 Hungary; 2 Doctoral School in Biology, University of Szeged, Szeged, Hungary; 3 Institute of Biophysics, Biological Research Centre, Szeged, Hungary; 4 Doctoral School in Theoretical Medicine, University of Szeged, Szeged, Hungary; 5 Doctoral School of Informatics, ELTE Eötvös Loránd University, Budapest, Hungary; 6 Department of Medical Chemistry, Faculty of Medicine, University of Szeged, Szeged, Hungary; 7 Institute of Medical Biology, University of Tromsø, Tromsø, Norway; 8 Institute for Molecular Medicine Finland (FIMM), University of Helsinki, Helsinki, Finland

**Keywords:** hHSPB1, Heat-shock protein, Astroglia, Cytokines, Ethanol toxicity, Microglia, Neuron, Neuroinflammation, Transgenic mice, Primary cells

## Abstract

**Background:**

Heat-shock protein B1 (HSPB1) is among the most well-known and versatile member of the evolutionarily conserved family of small heat-shock proteins. It has been implicated to serve a neuroprotective role against various neurological disorders via its modulatory activity on inflammation, yet its exact role in neuroinflammation is poorly understood. In order to shed light on the exact mechanism of inflammation modulation by HSPB1, we investigated the effect of HSPB1 on neuroinflammatory processes in an in vivo and in vitro model of acute brain injury.

**Methods:**

In this study, we used a transgenic mouse strain overexpressing the human HSPB1 protein. In the in vivo experiments, 7-day-old transgenic and wild-type mice were treated with ethanol. Apoptotic cells were detected using TUNEL assay. The mRNA and protein levels of cytokines and glial cell markers were examined using RT-PCR and immunohistochemistry in the brain. We also established primary neuronal, astrocyte, and microglial cultures which were subjected to cytokine and ethanol treatments. TNFα and hHSPB1 levels were measured from the supernates by ELISA, and intracellular hHSPB1 expression was analyzed using fluorescent immunohistochemistry.

**Results:**

Following ethanol treatment, the brains of hHSPB1-overexpressing mice showed a significantly higher mRNA level of pro-inflammatory cytokines (*Tnf*, *Il1b*), microglia (*Cd68*, *Arg1*), and astrocyte (*Gfap*) markers compared to wild-type brains. Microglial activation, and 1 week later, reactive astrogliosis was higher in certain brain areas of ethanol-treated transgenic mice compared to those of wild-types. Despite the remarkably high expression of pro-apoptotic *Tnf*, hHSPB1-overexpressing mice did not exhibit higher level of apoptosis. Our data suggest that intracellular hHSPB1, showing the highest level in primary astrocytes, was responsible for the inflammation-regulating effects. Microglia cells were the main source of TNFα in our model. Microglia isolated from hHSPB1-overexpressing mice showed a significantly higher release of TNFα compared to wild-type cells under inflammatory conditions.

**Conclusions:**

Our work provides novel in vivo evidence that hHSPB1 overexpression has a regulating effect on acute neuroinflammation by intensifying the expression of pro-inflammatory cytokines and enhancing glial cell activation, but not increasing neuronal apoptosis. These results suggest that hHSPB1 may play a complex role in the modulation of the ethanol-induced neuroinflammatory response.

**Supplementary Information:**

The online version contains supplementary material available at 10.1186/s12974-020-02070-2.

## Background

Neuroinflammation plays a central role in the pathophysiology of most neurological diseases. The activation of astro- and microglia cells, and their subsequent production of cytokines and chemokines are the main characteristics of neuroinflammation. Inflammation in the brain is primarily associated with the process of neurodegeneration, as it is initiated in order to aid the repair of the damaged brain area. However, in the case if it becomes dysregulated, a chronic inflammation could appear which further aggravates tissue damage. Therefore, the tight regulation of the neuroinflammatory response during neuronal tissue damage is crucial in preventing or at least in reducing the negative effects of chronic inflammation [1]. Heat-shock proteins (HSPs) could be one of the potential candidates for such an inflammation-regulating role, as they show a rapid induction under stress conditions, they promote cell survival under these conditions, and their modulatory effects on inflammatory processes are well described [2].

Heat-shock protein B1 (HSPB1, HSP27/25) is one of the most well-studied members of the evolutionarily conserved small heat-shock protein family. These ATP-independent chaperon proteins are characterized by low molecular weight between 16 and 40 kDa, and a conserved C-terminal α-crystallin domain. As molecular chaperones, small heat-shock proteins have an important role in maintaining the normal cellular protein homeostasis by binding to partially denatured proteins, thereby preventing their irreversible aggregation [3]. In the brain, HSPB1 is the most abundant in astrocytes under physiological conditions and it is even more elevated in response to various stress conditions, such as increased temperature or oxidative stress [4, 5]. Increased astroglial expression of HSPB1 was also detected after transient focal ischemia and in neurodegenerative disorders associated with pathological protein inclusions, such as tauopathies or Alzheimer’s disease [4, 6–8]. This line of observations suggests that HSPB1 might also play a role in reactive astrogliosis. Compared to astrocytes, HSPB1 is present in neurons to a lesser extent, although it was also described to be upregulated in pathological and under stress conditions [4, 9, 10]. In microglia, HSPB1 is present only in negligible amounts but following hyperthermia, it showed an elevated expression [11].

Besides its well-described role as a chaperon, HSPB1 is also associated with neuroinflammation through several mechanisms. HSPB1 can help to maintain the integrity of cytoskeletal networks through its association with various cytoskeletal filaments, such as the glial fibrillary acidic protein (GFAP), vimentin (Vim), or neurofilament [12, 13]. HSPB1 can also interact with several components of the apoptotic pathways and exert a strong anti-apoptotic function [14]. Moreover, it can modulate the release of cytokines from various cells; however, a multitude of effects can be observed in different studies in that regard. There has been an observation that described an HSPB1-dependent interleukin-10 (IL-10) increase and an associated anti-inflammatory response [15]. However, another study pointed towards a more pro-inflammatory role for HSPB1 as it was observed to induce the production of IL-8 and reduce the expression of anti-inflammatory transforming growth factor-β1 (TGF-β1) and the cluster of differentiation 40 (CD40) ligand in astrocytes in vitro [16]. In addition, HSPB1 is described to regulate the activation of the nuclear factor kappa B (NFκB) pathway. Recently, intracellular HSPB1 has been demonstrated to promote the degradation of IkappaB kinase-β resulting in an inactive state of NFκB and a subsequent decrease in tumor necrosis factor-α (TNFα) production in microglial cells [17]. However, in a macrophage culture, extracellular HSPB1 treatment was observed to activate the NFκB pathway via the degradation of the inhibitor of nuclear factor-kappa B-α (IκBα), thereby upregulating both pro- and anti-inflammatory factors, such as *IL1b*, *TNF*, IL-10, and the granulocyte-macrophage colony-stimulating factor [18]. Moreover, HSPB1 expressed in endothelial cells of the brain microvasculature contributes to an indirect protection against neuroinflammation. HSPB1 was described to ameliorate the impairment of the blood-brain barrier, and by this decreasing the influx of peripheral immune cells into the brain parenchyma after ischemic injury [19]. Overall, a growing number of evidence indicates that HSPB1 can affect the inflammation-related processes in several ways, but it seems to induce diverse effects depending on the cell type and the extra- or intracellular presence of the protein. Despite the diversity of the observed actions for HSPB1 in the central nervous system, the inflammation modulatory effects of this protein would require further clarification. In addition, there is a lack of comprehensive in vivo studies investigating the impact of HSPB1 on multiple components of inflammation within the CNS.

In this study, we aimed to test the hypothesis whether HSPB1 is involved in the regulation of cytokine expression, gliosis, and apoptosis in ethanol-induced acute neuroinflammation. We analyzed the inflammation-related processes using our previously generated human HSPB1 (hHSPB1)-overexpressing transgenic mouse strain [20], as well as using primary cell cultures isolated from the transgenic mouse model. Ethanol treatment is a suitable method for modeling the sterile inflammatory environment that serves as a central disease-promoting mechanism in many neurological disorders. In our in vivo experiments, we used early postnatal acute ethanol exposure because, due to the particularly high sensitivity of the developing brain to alcohol toxicity, even a single ethanol treatment leads to robust neurodegeneration, which is accompanied by neuroinflammatory processes, including cytokine release and glial cell activation [21, 22]. Accordingly, with this method, we were able to induce neuroinflammation rapidly in the brains of 7-day-old hHSPB1-overexpressing transgenic and wild-type mice and to investigate the possible immunomodulatory effects of hHSPB1 overexpression in this sterile inflammatory milieu. hHSPB1-overexpressing mice showed enhanced expression of pro-inflammatory cytokines *Tnf* and *Il1b* as well as increased immunoreactivity of astrocyte and microglial activation markers in response to ethanol-induced acute brain injury, whereas they exhibited a nonsignificant trend of decreased apoptosis. In addition, in vitro results showed that the release of hHSPB1 from the cells is minimal even after stress treatment. Therefore, we suggest that the intracellular form of hHSPB1 is responsible for the observed inflammation-regulating effects, showing a significantly increased expression in primary astrocytes after cytokine and ethanol treatment. Moreover, microglia seemed to be the main source of pro-inflammatory cytokines in vitro, since the highest TNFα response to cytokine treatment was detected in the supernates from microglia cultures from hHSPB1 transgenic animals. Taken together, we found that the ethanol-induced neuroinflammatory processes were aggravated in our HSPB1-overexpressing mice including the pro-inflammatory cytokine expression and glial cell activation, while we could not detect the further increase of the inflammation-related neuronal damage in these animals, suggesting that HSPB1 has a complex role in the regulation of ethanol-induced acute inflammation.

## Methods

### In vivo experiments

#### Animals

The study has been carried out according to the EU Directive 2010/63/EU and was approved by the regional National Food Chain Safety Agency and Animal Health Directorate (Csongrad-county, Hungary) under the project license XVI/4136/2014. Mice were housed in groups of two to three under standard conditions (24 °C, 12 h of light-dark cycle) with food and water available ad libitum. Seven-day-old hHSPB1 transgenic mice and wild-type littermates were used for the studies. The hHSPB1-overexpressing transgenic line was previously established by our group on a homogenous C57BL/6 genetic background [20].

#### Ethanol treatment

Neurodegeneration was induced in 7-day-old wild-type and hHSPB1-overexpressing transgenic mice by the subcutaneous injection of 20% ethanol solution as described earlier in Ikonomidou et al. [21]. Ethanol was administered in two equal doses of 2.5 g/kg body weight within 2 h (at zero and at the 2nd hour), resulting in a total dose of 5 g/kg. Control pups were treated with physiological saline. After cervical dislocation and decapitation, brains were removed at 7 h/24 h/1 week after the second injection and processed as follows: the dissected brains were embedded in optimal cutting temperature (OCT) compound (Tissue-Tek, Sakura Finetek, CA, USA) for cryostat sectioning and to carry out the terminal deoxynucleotidyl transferase dUTP nick end labeling (TUNEL) assay; for immunohistochemistry, brains were immersion-fixed in 3% paraformaldehyde (PFA)-0.1 M phosphate buffer solution; the brains were further homogenized in AccuZol Reagent for RNA isolation or in radioimmunoprecipitation assay (RIPA) buffer for protein isolation. At the same time, small tail biopsies were taken for subsequent genomic DNA isolation and genotype determination.

#### hHSPB1 Western blot analysis

Protein level of transgenic human HSPB1 was determined from whole-brain homogenates of 7-day-old mice (*n* = 3 mice per group). Brains were homogenized in 1 ml RIPA buffer containing 2 mM phenylmethylsulfonyl fluoride and 2 mM N-ethylmaleimide. After centrifugation (13,000*g* for 25 min at 4 °C), protein concentrations were measured in the supernates at 280 nm using a NanoDropND-1000 spectrophotometer (Nanodrop, DE, USA). Five milligram per milliliter samples were mixed with sample buffer containing sodium dodecyl sulfate (SDS), glycerol, β-mercaptoethanol, and bromophenol blue, and incubated at 95 °C for 5 min. Protein samples (50 μg) were loaded into a 15% SDS-polyacrylamide gel and samples were run at 80 V for 2 h in 1× SDS-PAGE running buffer. Then, proteins were transferred to polyvinylidene difluoride membranes (Pall Corporation, NY, USA), were submerged in 1× transfer buffer, and run at 200 mA for 1 h on ice. Subsequently, membranes were incubated in phosphate-buffered saline with Tween 20 (PBST) containing 5% nonfat milk powder for 1 h at room temperature. The following primary antibodies were used in the study: rabbit anti-hHSPB1 (O/N at 4 °C, Stressgen - Enzo Life Sciences, NY, USA, no cross-reactivity with mouse HSPB1) and rabbit anti-actin (2 h at room temperature, Sigma-Aldrich Ltd, Budapest, Hungary). The secondary antibody used was horseradish peroxidase–conjugated goat anti-rabbit (Jackson ImmunoResearch Europe Ltd., Cambridgeshire, UK) (see Table S1 for details of antibodies). Afterwards, membranes were incubated with a chemiluminescent detection reagent (Luminata Forte Western HRP Substrate, Merck Millipore, MA, USA) for 2 min and blots were developed manually on X-ray films. Quantification of the results was performed using the open-access ImageJ software.

#### mRNA isolation and reverse transcription

mRNA was isolated from whole brains of the mice. Homogenized tissue was mixed with AccuZol Reagent (Bioneer, South Korea), by adding 1 ml to each tissue sample. Subsequently, 200 μl chloroform was added to the samples followed by incubation on ice for 5 min. Phases were separated by centrifugation at 13,000*g* for 15 min at 4 °C, and the aqueous phase was separated from the organic phase from where the RNA was precipitated with 100% isopropyl alcohol during incubation for 10 min at − 20 °C. After centrifugation for 10 min at 13,000*g* at 4 °C, the RNA pellets were washed with 80% ethanol and the samples were centrifuged for 5 min at 13,000*g* at 4 °C. The RNA pellets were dissolved in RNase-free water and cleaned by using Nucleo Spin mRNA Clean-Up columns (Macherey-Nagel, Düren, Germany). Samples were treated with DNase and were eluted from the membrane with RNase-free water. Concentrations of the samples were measured at 230 nm using a spectrophotometer (NanoDrop ND-1000, Nanodrop, DE, USA). mRNA samples were converted to cDNA using reverse transcription (High Capacity cDNA Reverse Transcription Kit, Applied Biosystems, CA, USA) according to the manufacturer’s instructions.

#### Real-time PCR analysis

Real-time (RT)-PCR was performed to analyze the level of hHSPB1, cytokines, and glial markers. Gene-specific primers are listed in Table S2. Each reaction was performed in a total volume of 20 μl containing 10 μl of Power SYBR Green PCR Master Mix (Applied Biosystems, CA, USA), 1 μl of 5 pmol/μl primer mix (forward + reverse), and 9 μl of cDNA sample. The amplification was carried out on a RotorGene 3000 instrument (Corbett Research, Australia) with the following cycling parameters: heat activation at 95 °C for 10 min; followed by 45 cycles of denaturation at 95 °C for 15 s, annealing at 56 °C for 15 s, and extension at 60 °C for 40 s. Fluorescent signals were collected after each extension step at 72 °C and at the end, the registration of the melting curve was performed between 50 and 95 °C. Expression level of the target genes was normalized to an endogenous control (glyceraldehyde 3-phosphate dehydrogenase, *Gapdh*). Relative expression of the target genes compared to the untreated wild-type animals was calculated using the ΔΔCt method (*n* = 8 mice per group at 24 h; *n* = 3 mice per group at 7 h and 1 week).

#### TUNEL assay

To assess the level of apoptotic cell death, TUNEL assay was used, which labels the fragmented DNA. Brains were embedded in OCT compound, then 10-μm sagittal frozen sections were prepared. Sections were post-fixed in 4% paraformaldehyde in phosphate-buffered saline (PBS) solution (pH = 7.4) for 20 min. Following a 30-min PBS rinse, sections were blocked in 3% H_2_O_2_ in methanol for 10 min, then they were permeabilized with a solution of 0.1% Triton X-100 and incubated in 0.1% sodium citrate for 2 min. After washing with PBS, 50 μl of TUNEL reaction mixture was added to each sample (In situ Cell Death Detection Kit, POD, Roche Applied Science, IN, USA) and the sections were incubated for 1 h at 37 °C in a humidified atmosphere. Under the same circumstances, the sections were incubated with Converter-POD solution for 30 min, then washed in PBS, followed by a 10-min signal conversion with a peroxidase reagent. The red apoptotic cells were detected under a light microscope. For quantitative comparison, the TUNEL-positive cells were counted in fifteen fields of view of each brain section (*n* = 6 mice per group).

#### Fluorescent immunohistochemistry

Double immunostaining was performed to study the expression pattern of hHSPB1. After washing in PBS, 30-μm-thick frozen sagittal brain sections were permeabilized and blocked with 0.2% Triton X-100 and 3% bovine serum albumin (BSA) in PBS for 1 h at room temperature. Then the sections were incubated overnight at 4 °C with the following primary antibodies: goat anti-IBA1 (ionized calcium–binding adaptor molecule 1, Abcam, Cambridge, UK), mouse anti-GFAP (Sigma-Aldrich Ltd, Budapest, Hungary), mouse anti-NEUN (neuronal nuclei, Merck Millipore, MA, USA), and rabbit anti-hHSPB1. Appropriate secondary antibodies were applied for 2 h: Alexa Fluor-488–conjugated rabbit anti-goat (Jackson ImmunoResearch Europe Ltd., Cambridgeshire, UK); FITC-conjugated goat anti-mouse (Sigma-Aldrich Ltd, Budapest, Hungary); Alexa Fluor-647–conjugated goat anti-rabbit (Thermo Fisher Scientific, MA, USA) (Table S1). Cell nuclei were counterstained with 4′,6-diamidino-2-phenylindole (DAPI) (Sigma-Aldrich Ltd, Budapest, Hungary), at the concentration of 0.5 μg/ml for 5 min. Immunostainings were examined with a confocal laser scanning microscope (Olympus Fluoview FV1000, Olympus Life Science Europa GmbH, Hamburg, Germany).

#### Peroxidase immunohistochemistry

To analyze the activation of glial cells, brain sections were labeled with microglia and astrocyte markers. The brain region–specific expression pattern of the transgenic hHSPB1 protein was also examined with this method. First, the brain sections were treated with 20% methanol and 3% H_2_O_2_ for 15 min to deactivate endogenous peroxidases. The sections were washed in PBS then blocked for 2 h at room temperature using 2% normal rabbit or horse serum and 0.3% BSA in PBS. Then the sections were incubated with rabbit anti-hHSPB1, mouse anti-GFAP, or goat anti-IBA1 primary antibodies overnight at 4 °C. Subsequently, sections were incubated with the appropriate secondary antibodies: peroxidase-labeled goat anti-rabbit, peroxidase-conjugated rabbit anti-mouse (Chemicon-Merck Millipore, MA, USA), or biotinylated donkey anti-goat (Jackson ImmunoResearch Europe Ltd., Cambridgeshire, UK) for 2 h (Table S1). The sections were then incubated with the chromogen substrate, 3,3′-diaminobenzidine (DAB) (Sigma-Aldrich Ltd, Budapest, Hungary) at the concentration of 10 mg/ml for 15 min, and the solution was supplemented with 1% nickel chloride. For IBA1 staining, an avidin-biotin (VECTASTAIN Elite ABC Peroxidase Kit, Vector Laboratories, CA, USA) treatment was applied for 2 h at room temperature as a signal amplification step before visualizing the immunoreaction with DAB. The immunostained sections were digitally scanned using a slide scanner (Mirax Midi, 3DHistech Ltd., Budapest, Hungary) (*n* = 3 mice per group and 3 sections per animal). Images were analyzed with the Pannoramic Viewer 1.15.4; CaseViewer 2.1 and QuantCenter; and HistoQuant module softwares (3DHistech Ltd., Budapest, Hungary). Each region of interest was manually outlined, then followed by automated detection of relative areas of immunopositivity. Results are given in percentage of the immunopositive areas compared to the outlined areas (relative area).

#### Automated analysis of microglial morphology with deep learning approach

##### Morphological categorization

The activity state of microglia can be well characterized by their morphology, based on which microglia were classified into three subtypes: ramified (first class), intermediate (second class), and amoeboid (third class) [23, 24]. The ramified morphology of resting microglia is characterized by a small cell body and branching, long, thin processes extending far from the cell body (Fig. S1a). Cells classified into intermediate and amoeboid groups can be considered activated. The former is characterized by having an enlarged cell body and numerous thick, shortened processes which are still branching, while the latter is characterized by a large cell body which is completely round or has a few very short and thick primary processes (Fig. S1b).

##### Annotation procedure

Manual annotations were performed using the AnnotatorJ software [25]. In total, 5883 cells on 232 images were annotated independently by two experts. Each annotated object in the set contains an associated class label and a bounding rectangle that was drawn around it. The total number of classes is 3. The number of the annotated cells in the first class was 2961 and 1461-1461 in the second and third. Rectangle size can vary and they can intersect each other.

For performance evaluation, 30 images with a total of ~ 800 cells were selected. This validation set was annotated by both of the experts. Performing this annotation at two different time points, we measured the intraexpert accuracies.

##### Evaluation with deep learning

Training the model: *pytorch* [26], a Python-based deep learning package was used to detect microglia (we used Python 3.6). We trained multiple models based on two different architectures, namely the *yolov3-SPP* [27] and *CSPResNext50-PANet-SPP* [28]. To make the training robust, the training data was augmented using random affine transformations. We were using ADAM (Adaptive Moment Estimation [29]) optimizer with a base learning rate of 0.001. Because for such data there are no pretrained weights available, we used random initialization. The number of epochs for each setup was 2000. This resulted in 0.594 as the best mAP (mean average precision) for *yolov3-SPP* and 0.593 as the best mAP for *CSPResNext50-PANet-SPP*. The training was performed on a GPU cluster with a 2.10 GHz Intel(R) Xeon(R) ES-2620 CPU, 32 GB memory, and NVIDIA Titan Xp graphics card and for both cases took roughly 72 h.

##### Microglia detection with classification

We selected the *yolov3-SPP* model for the evaluation part because it resulted both statistically and visually in better results. To evaluate the performance of the proposed deep learning model, we measured precision, recall, and F1 score of two different annotators. The object matching between the detection and classification results and the ground truth was done manually by an expert. We considered a bounding box detection to be correct (true positive, TP) when it fully contained a cell; otherwise, it was considered as false positive (FP). When the detection algorithm was not able to find an object that was listed in the ground truth, that sample was treated as false negative (FN). Based on these conditions, we calculated the precision (P) = TP / (TP + FP); recall (R) = TP / (TP + FN); F1 score (F1) = 2 * P * R / (P + R); and detection accuracy (DA) = TP / (TP + FP + FN).

To inspect the accuracy of the classification part, we have checked all the images selected for the evaluation. As the detection accuracy was already calculated, we supervised the predicted class labels for each object. Based on the correctly (C) and incorrectly (IC) predicted class labels, we calculated the classification accuracy (CA) = (C) / (C + IC) (Fig. S1c).

For the final detection result, we evaluated four different comparisons. First, we measured self-accuracy of the human experts. This involves intra- and interexpert accuracies. We measured intraexpert accuracy by multiple annotations of the same images made by the same expert. We did this by annotating the same images with 2 weeks time difference. The intraexpert accuracies resulted in 97.4% accuracy (precision = 0.985, recall = 0.989, F1 score = 0.987) and 97.8% accuracy (precision =  0.984, recall = 0.993, F1 score = 0.988). Meanwhile, interexpert accuracy is the comparison of the annotations made by two experts on the same test set. This observation has reached 92.9% accuracy (precision = 0.949, recall = 0.977, F1 score = 0.963). In these cases, we used the test set made by the first annotator as the ground truth. Finally, we compared the ground truth to the predicted bounding boxes. This performed almost as well as the annotators and reached a surprising 94.4% accuracy (precision = 0.966, recall = 0.977, F1 score = 0.971) (Fig. S1d).

For the final classification result, we evaluated four different comparisons, following the same scheme as for the detection part. The intraexpert accuracies reached 97% accuracy and 98.1% accuracy. The interexpert accuracy resulted in 90.5% accuracy. And the final comparison achieved 90.1% accuracy.

### In vitro experiments

#### Preparation of primary cultures

The isolation of primary neurons was carried out according to Pacifici and Peruzzi [30] with some modifications. Primary cortical neurons were obtained from hHSPB1 transgenic mice and wild-type littermates on the 17th embryonic day. Briefly, embryos were washed in cold dissection medium, then the whole brain was extracted and was placed into an ice-cold Hibernate E medium (BrainBits, IL, USA). Cerebral cortices were isolated, while meninges were removed from the surface, and cortices were washed 3 times with Hibernate E, then were enzymatically digested with TrypLE (Gibco, Life Technologies, CA, USA) at 37 °C for 10 min. Next, the digested cortices were washed 3 times with Hibernate E and were homogenized in Neurobasal/B27 complete medium (Gibco, Life Technologies, CA, USA) containing 200 mM Glutamax (Gibco, Life Technologies, CA, USA) with 18-G and 20-G needles (Braun, Germany). Finally, the suspension was diluted with Neurobasal/B27 complete medium, and cells were counted using Countess Automated Cell Counter (Thermo Fisher Scientific, MA, USA). Cells were plated on poly-d-lysine-coated 96-well plates at the density of 2 × 10^4^ cells/well. Primary neurons were cultured in an incubator with 5% CO_2_ and 37 °C for 5 days.

Primary mouse glial cells were isolated and cultured as described in Lenárt et al. [31]. Briefly, forebrains without the cerebellum and the bulbus olfactorius were obtained from 4-day-old wild-type and hHSPB1 transgenic mice and placed into ice-cold PBS. Meninges were removed from the surface of the brains by using a fine forceps and sterile filter paper. Little pieces of cortices were pipetted to 50-ml tubes and then the tissue was mechanically dissociated using a long and thin needle (21G 4 ¾, Braun, Germany). Isolated cells were plated onto uncoated T25 flasks (Corning Costar Co., MA, USA) and cultured in low-glucose Dulbecco’s modified Eagle’s medium (Gibco, Life Technologies, CA, USA) containing 10% fetal bovine serum (Sera Plus, Pan Biotech, Aidenbach, Germany) and gentamycin (Sigma-Aldrich Ltd, Budapest, Hungary) at the concentration of 50 μg/ml and were cultured until confluency before used for experiments. Medium was changed every 2 days.

Microglial cells were isolated from the surface of primary astroglia cultures [32]. Here astroglia were not passaged after reaching confluency but kept in the flask for longer periods. After 10 days, microglia cells migrate to the top of the confluent astroglia layer and start to round up on the surface. After gently tapping the flasks, the detaching microglia cells were collected and plated in astroglia culture medium onto poly-l-lysine-coated 96-well plates with a cell density of 2 × 10^4^ cells/well. With this method, additional microglia cells were collected every second day for up to a week from the surface of the glial layer. Half of the medium was changed every day. Microglia cells received 50% glia cell–conditioned medium from day 3 and were cultured for 7 days before treatments.

#### Assessment of the purity of the isolated primary cell cultures by immunostaining

In order to determine the purity of primary neuron, astroglia, and microglia cultures, cells were passaged either to thin-bottom 96-well plates (Corning Costar Co., MA, USA) or to coverslips (VWR, PA, USA) and stained for specific markers. Glial cells and microglia were cultured on poly-l-lysine-coated glass coverslips and in 96-well plates along with neurons. Following the regular permeabilization and blocking steps (for this, see Methods’ section “Fluorescent immunostaining”), cells were stained for specific markers. To evaluate the purity of the neuronal cultures, cells were double-labeled for neuronal marker microtubule–associated protein-2 (chicken anti-MAP2, Abcam, Cambridge, UK) and astrocyte marker GFAP (mouse anti-GFAP). Primary isolated astroglia cultures can be contaminated by microglia cells and vice versa; therefore, to assess the level of astroglia and microglia purity, we stained for GFAP and for the microglial marker IBA1 (goat anti-IBA1) (Table S1). The following secondary antibodies were used: Alexa Fluor-594–conjugated donkey anti-chicken (Jackson ImmunoResearch Europe Ltd., Cambridgeshire, UK); Alexa Fluor-488–conjugated donkey anti-mouse (Life Technologies, Invitrogen, USA), Alexa Fluor-488–conjugated donkey anti-goat (Life Technologies, Invitrogen, USA), Dylight 549–conjugated goat anti-mouse (Jackson ImmunoResearch Europe Ltd., Cambridgeshire, UK) (Table S1). After mounting the samples (Fluoromount-G; Southern Biotech, AL, USA), pictures were taken at random positions (at least 3 images/sample), using fluorescent confocal laser scanning microscopes: Olympus Fluoview FV1000 (Olympus Life Science Europa GmbH, Germany) and Leica SP8 Confocal Microscope (Leica Microsystems, Germany). Cell nuclei were counted for both stainings and an image quantitation was performed where percentages of the marker positive cells were given normalized to the total cell number (*n* = 3–11).

#### Treatments and collection of supernates

Primary neurons were treated 5 days after the isolation with human TNFα and IL-1β (10 ng/ml each) and 200 mM EtOH. The treatments on primary neurons were performed in 96-well plates in 50 μl/well Neurobasal/B27 complete medium (Gibco, Life Technologies, CA, USA). After 24 h of treatment, cell culture supernates were collected, an 3-3 wells were pooled, centrifuged at 510*g* to remove any contaminating cells or debris, and then stored at − 80 °C. Meanwhile, cells were fixed with 4% PFA for 20 min and then stored in 1× PBS at 4 °C for immunofluorescent staining.

All treatments and supernate collection from primary astroglia and microglia cells were performed similarly except that a lower concentration of EtOH (50 mM) was used in glial cell culture medium. After the treatment, cell culture supernates were collected and stored at − 80 °C. Cells were fixed with 3% PFA for 15 min at room temperature and then stored in PBS containing 0.1% sodium azide at 4 °C for immunofluorescent staining.

#### Fluorescent immunostaining

To analyze the expression pattern of hHSPB1, PFA-fixed primary neuronal cells were permeabilized with 0.1% Triton X-100 for 10 min. Cells were then blocked with 3% BSA in PBS for 1 h and were incubated overnight with primary antibodies: chicken anti-MAP2 and rabbit anti-hHSPB1. Subsequently, the cells were incubated with secondary antibodies: Alexa Fluor-594–conjugated donkey anti-chicken and Alexa Fluor-488–conjugated goat anti-rabbit (Thermo Fisher Scientific, MA, USA) for 45 min (Table S1). Cell nuclei were labeled with DAPI (Sigma-Aldrich Ltd, Budapest, Hungary) at 0.5 μg/ml for 5 min. The stainings were examined with a Leica SP8 Confocal Microscope (Leica Microsystems, Germany).

To further assess the hHSPB1 expression, microglia and astroglia cells were co-stained for their corresponding markers and for hHSPB1*.* After PFA fixation, permeabilization was carried out with 0.2% Triton X-100 in PBS for 10 min, followed by blocking with 2% normal horse serum or 5% normal goat serum. Primary antibodies were incubated overnight: goat anti-IBA1, mouse anti-GFAP, rabbit anti-hHSPB1. Incubation with secondary antibodies Alexa Fluor-488–labeled donkey anti-goat, Cy3-labeled sheep anti-rabbit (Sigma-Aldrich Ltd, Budapest, Hungary), Dylight A488–conjugated goat anti-rabbit (Jackson ImmunoResearch Europe Ltd., Cambridgeshire, UK), and Dylight 549–conjugated goat anti-mouse lasted for 1 h. Hoechst dye 33342 was used for nucleus staining (Table S1). After mounting the samples (Fluoromount-G; Southern Biotech, AL, USA), staining was examined by Olympus Fluoview FV1000 confocal laser scanning microscope (Olympus Life Science Europa GmbH, Hamburg, Germany).

The images were analyzed using the Matlab software (MathWorks, Natick, MA, USA). An average threshold was calculated for each staining using the images from the transgenic and wild-type animals. The binary images were determined by using the corresponding average threshold. Objects with a size of less than 4 pixels were eliminated to reduce any false structures. The colocalization was received by taking overlapping structures of the corresponding binary images. The overall intensities were calculated using the final binary images as masks on the original grayscale images. The intensity values gained from astrocytes were normalized to the pixel numbers from where the intensity data was collected (*n* = 9–12 for GFAP in TG astrocytes; *n* = 14–15 for GFAP in WT astrocytes; *n* = 10–11 for hHSPB1 in TG astrocytes). Due to the presence of astrocytes in neuronal culture, which also highly express the transgene, HSPB1 intensity values were obtained from neurons and normalized to the pixel number of neuronal marker MAP2 (*n* = 12).

#### Enzyme-linked immunosorbent assay analysis of cell culture supernates

Concentrations of released hHSPB1 and TNFα in the supernate of all three primary isolated cell cultures were quantified using human HSPB1 ELISA kit (Enzo Life Sciences, NY, USA) and mouse TNF alpha ELISA kit (Life Technologies, Invitrogen, USA). Briefly, cell culture supernates were collected and additional sample diluents were added. For all cell types, samples from multiple experiments were tested to create biological and technical parallels (*n* = 2–3). We used 47 μl sample/well for the mouse TNFα assay, and 95 μl sample/well for the hHSPB1 assay. After the addition of supernates to the wells, the assay was carried out according to the manufacturer’s instructions.

#### Statistical analysis

All data obtained in this experiment are expressed as mean ± SEM. Statistical analysis was performed by two-way analysis of variance (2-way ANOVA) followed by Tukey post hoc test using the OriginPro8 software (OriginLab, MA, USA) and unpaired two-tailed *t* test using GraphPad Prism software (GraphPad Software Inc., San Diego, CA, USA). Data were considered statistically significant at *p* < 0.05.

## Results

### Expression pattern of transgenic human HSPB1 in the mouse brain

**Fig. 1:**
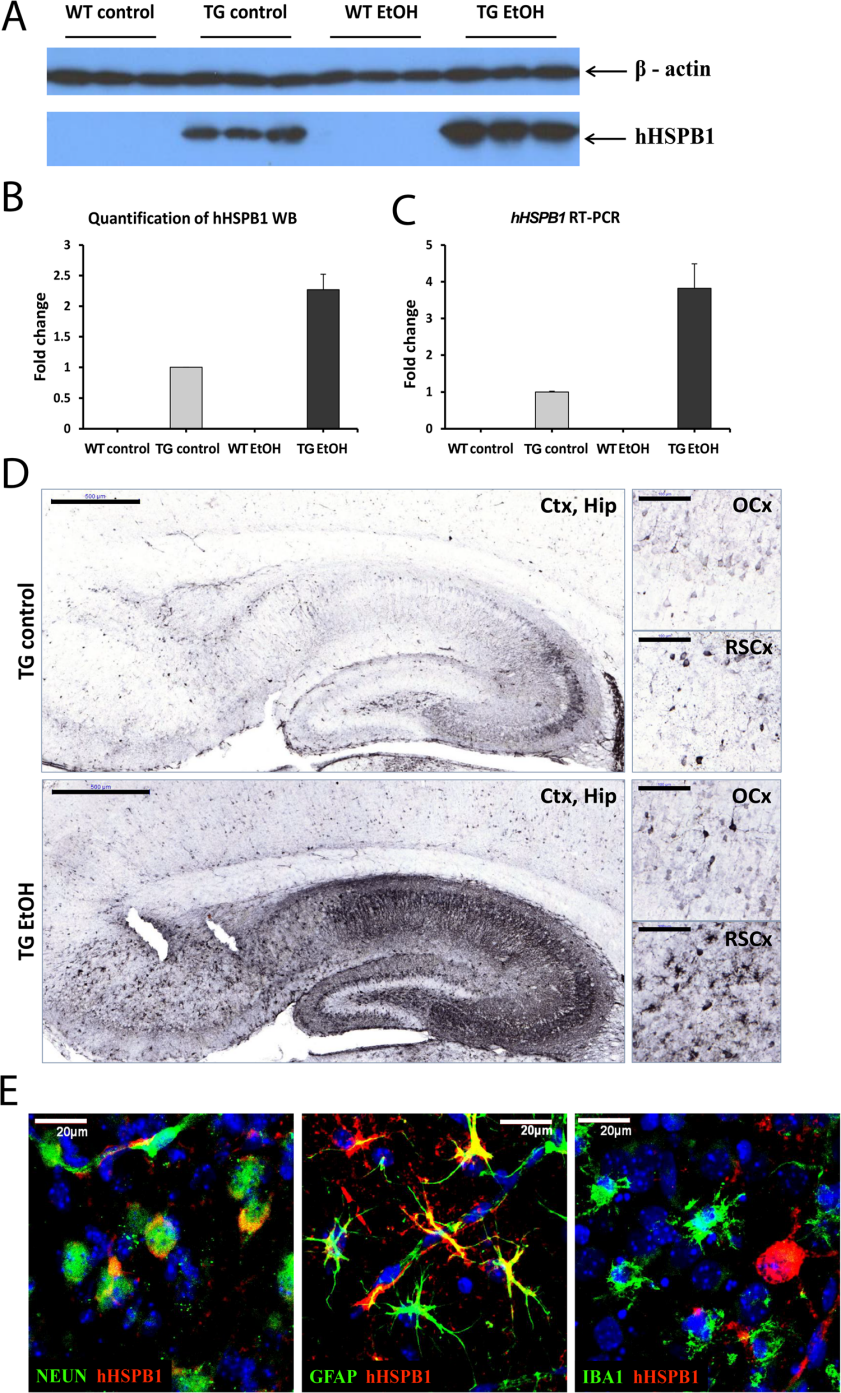
Transgene expression analysis in the brain. **a** hHSPB1 protein expression level was measured in whole-brain homogenates 24 h after ethanol treatment using Western blot. Mouse β-actin was used as an internal control. **b** Quantification of the results was performed using the open-source ImageJ software. Fold changes were correlated with the transgenic control group. Data are represented as mean ± SEM; *n* = 3 mice per group. **c** Relative expression of the transgenic *hHSPB1* 24 h after ethanol treatment was studied in the brain using RT-PCR. Fold changes were correlated with the transgenic control (nontreated) group. Values are presented as mean ± SEM; *n* = 8 mice per group. **d** hHSPB1 immunolabeling on sagittal brain sections of saline- and ethanol-treated transgenic mice 24 h after treatment. Scale bar: 500 μm (Ctx, Hip) and 100 μm (OCx and RSCx). Ctx cortex, Hip hippocampus, RSCx retrosplenial cortex, OCx occipital cortex. **e** Fluorescent double immunohistochemistry on sagittal brain sections showing cell type–specific expression of the transgene in the cortical region. Scale bar: 20 μm. Red: hHSPB1; green: IBA1/NEUN/GFAP; blue: DAPI

The expression of the transgenic hHSPB1 was studied in the brain of 7-day-old mice using various methods. Whole-brain homogenates were analyzed by Western blotting (Fig. 1a, b) and RT-PCR (Fig. 1c). High expression of transgenic hHSPB1 was detected in transgenic brains both at the protein and mRNA levels. hHSPB1 expression increased 24 h after ethanol treatment, especially in the hippocampal region and in the retrosplenial cortex, as shown by our immunohistochemical analysis (Fig. 1d). The cell-specific expression pattern of the transgene was also monitored using NEUN-hHSPB1, GFAP-hHSPB1, and IBA1-hHSPB1 fluorescent double immunostainings (Fig. 1e). The NEUN-hHSPB1 double immunostaining revealed the presence of the transgenic hHSPB1 protein in neuronal cells. The GFAP immunolabeling colocalized with hHSPB1, indicating that astrocytes also expressed the transgene. IBA1 immunoreactive microglia, however, showed no remarkable colocalization with hHSPB1.

### Differences in the gene expression of pro- and anti-inflammatory cytokines in hHSPB1 transgenic and wild-type mouse brains

**Fig. 2:**
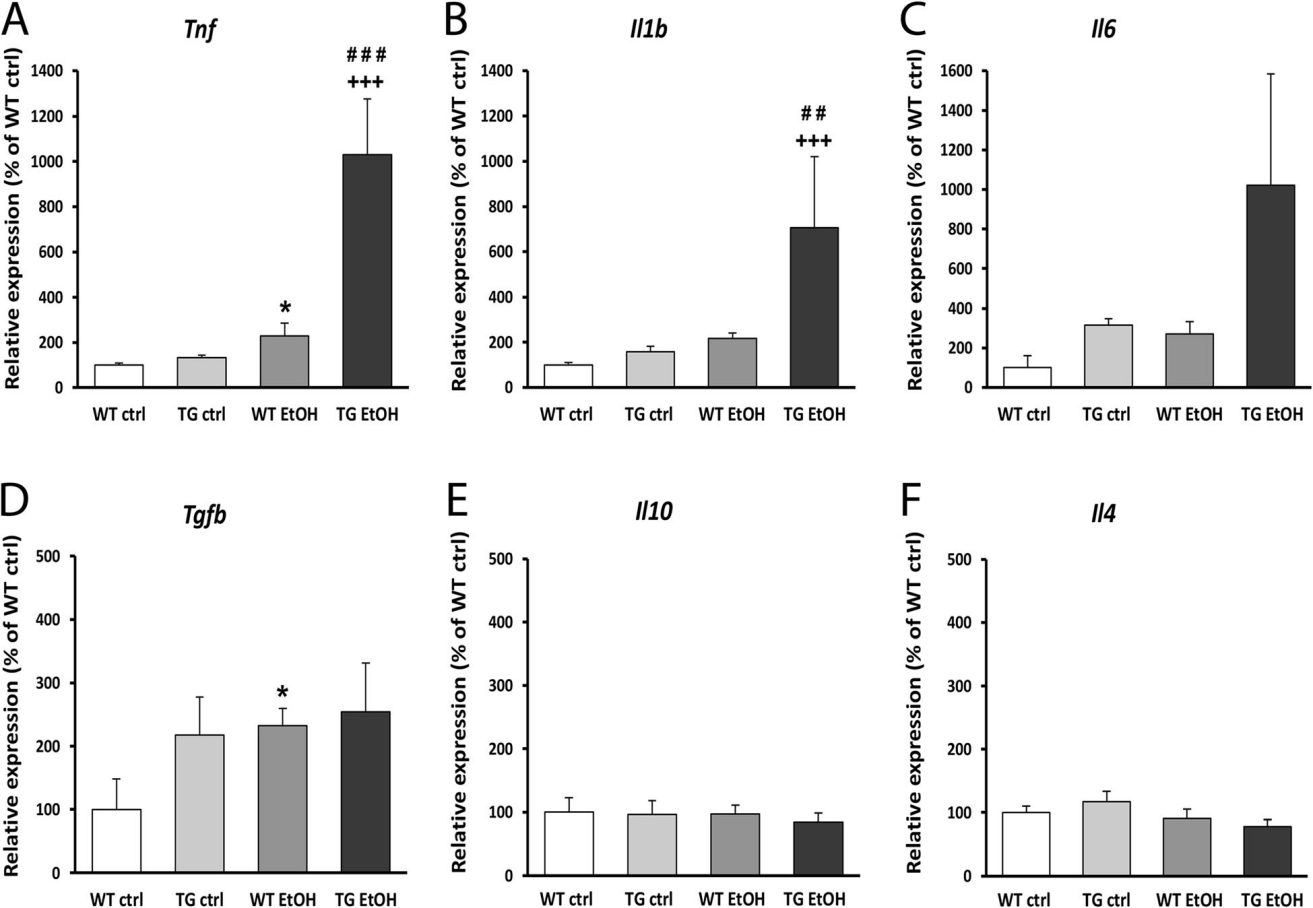
Gene expression analysis of inflammatory cytokines and glial activation markers in the brain. Relative expression of **a** *Tnf*, **b** *Il1b*, **c** *Il6*, **d** *Tgfb*, **e** *Il10*, and **f** *Il4* was studied in the brain of hHSPB1-overexpressing and wild-type mice 24 h after ethanol treatment using RT-PCR. Relative expression was correlated with the wild-type control group (100%). Values are presented as mean ± SEM; *n* = 8 mice per group. Asterisk indicates wild-type EtOH vs wild-type control group; plus sign indicates transgenic EtOH vs transgenic control group; hashtag indicates transgenic EtOH vs wild-type EtOH group. Statistical analysis: 2-way ANOVA followed by Tukey post hoc test. *p < 0.05; ^##^*p* < 0.01; ^###/+++^*p* < 0.001

The expression level of cytokines as molecular markers of inflammation was studied using semiquantitative RT-PCR at different time points after ethanol treatment. In nontreated transgenic mice, hHSPB1 overexpression alone did not affect significantly the mRNA level of the studied pro-inflammatory cytokines, whereas it contributed to a marked increase in gene expression after ethanol treatment. In response to ethanol, the level of *Tnf* mRNA was elevated as early as 7 h, showing a threefold (297%) increase in wild-type and a fourfold (413%) increase in hHSPB1-overexpressing animals compared to saline-treated controls (Fig. S2a). One day later, *Tnf* expression started to decrease in the wild-type animals but still maintained a twofold (229%) increase compared to the nontreated controls (Fig. 2a). However, in transgenic animals, we detected a further elevation in the level of *Tnf* mRNA, reaching a tenfold (1031%) increase in expression compared to untreated wild-types (Fig. 2a). Similar changes were found in the expression level of *Il1b*: it also increased in response to the ethanol treatment; however, this had happened at a later time point. One day after the treatment, it showed a twofold increase in the wild-type animals, while in the transgenic ones, we detected a much higher, a more than sevenfold increase (706%, Fig. 2b). Another inflammation-inducing cytokine, *Il6*, also showed a similar, but nonsignificant trend towards increased expression in ethanol-treated transgenic mice (Fig. 2c). Contrarily, in the case of anti-inflammatory cytokines, we observed changes only in the mRNA level of *Tgfb*, whereas the expression levels of *Il10* and *Il4* remained unchanged in response to hHSPB1 overexpression as well as after ethanol treatment (Fig. 2d–f). In the nontreated group, the expression level of the *Tgfb* was twice as high in hHSPB1-overexpressing animals as in wild-type ones 24 h after saline injection (Fig. 2d). One day after ethanol administration, the expression level of *Tgfb* showed a significant increase in wild-type animals (233%), whereas in transgenic mice, no further increase was detected. After 1 week, the gene expression levels of the investigated cytokines decreased to their original levels both in the wild-type and in the transgenic brains (Fig. S2a-c).

### Altered microglial activation in hHSPB1 transgenic brains

**Fig. 3:**
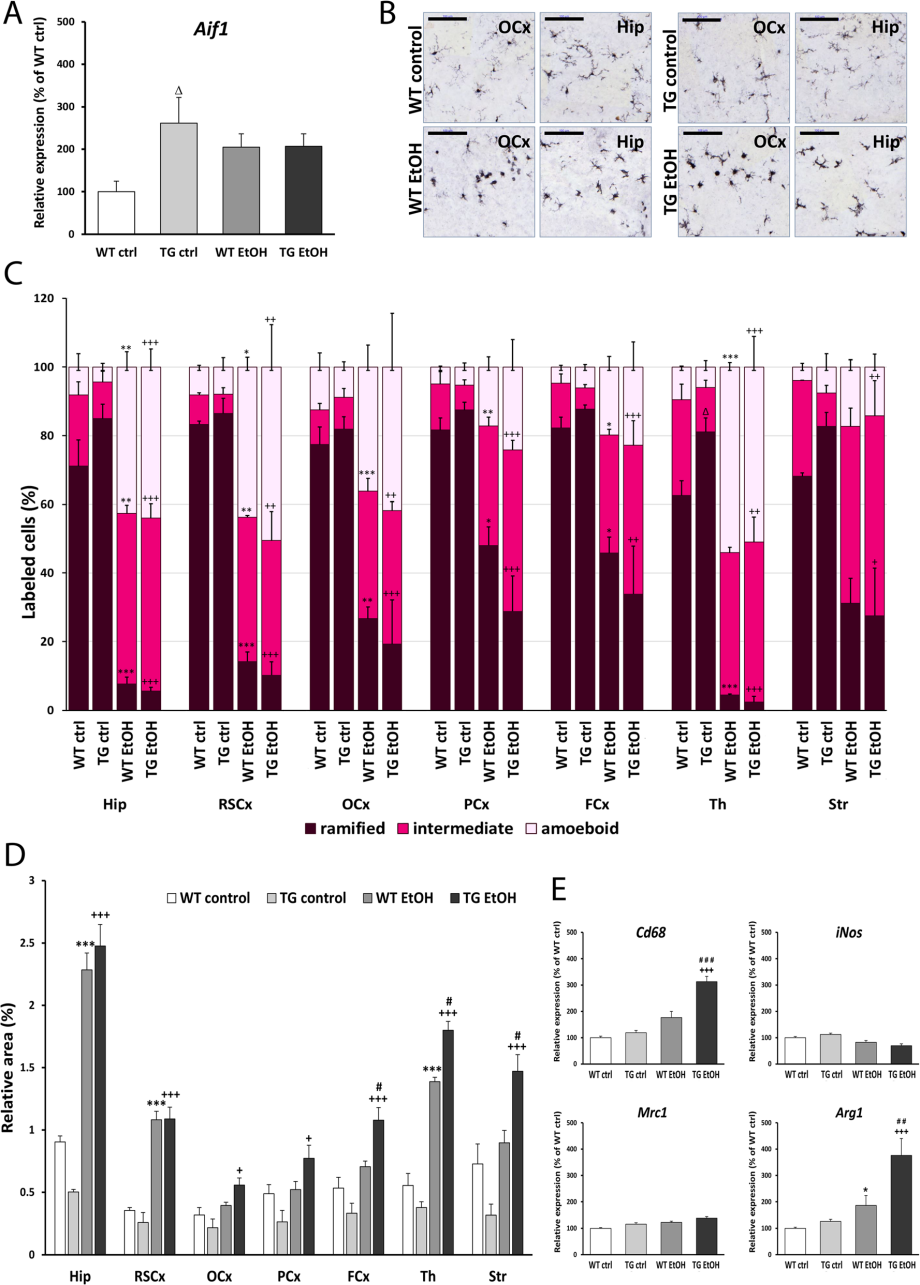
Activation of microglia 24 h after ethanol treatment in the brain of transgenic and wild-type mice. **a** Relative expression of *Aif1* in the brain of hHSPB1-overexpressing and wild-type mice 24 h after ethanol treatment. Relative expression was correlated with the wild-type control group (100%). *n* = 8 mice per group. **b** Morphological changes of microglia 24 h after EtOH treatment on sagittal brain sections of hHSPB1-overexpressing and wild-type mice. Microglia with different morphologies were visualized with IBA1 immunostaining using the peroxidase method. Scale bar: 100 μm (OCx and Hip). **c** Proportions of IBA1+ microglia in different brain regions according to their morphology. **d** Quantification of IBA1 immunoreactive areas. Results are given in percentage of the immunopositive areas compared to the outlined brain areas (relative area). *n* = 3 mice per group, 3 sections per animal. **e** Relative expression of M1 microglia markers (*Cd68*, *iNos*) and M2 microglia markers (*Mrc1*, *Arg1*) in the brain of hHSPB1-overexpressing and wild-type mice 24 h after ethanol treatment. Relative expression was correlated with the wild-type control group (100%). *n* = 8 mice per group. Values are presented as mean ± SEM. Asterisk indicates wild-type EtOH group vs wild-type control group; plus sign indicates transgenic EtOH group vs transgenic control group; hashtag indicates transgenic EtOH group vs wild-type EtOH group; white triangle indicates transgenic control vs wild-type control group. Statistical analysis: 2-way ANOVA followed by Tukey post hoc test. *^/#/+/Δ^*p* < 0.05; **^/##/++^*p* < 0.01; ***^/###/+++^*p* < 0.001. Ctx cortex, Hip hippocampus, RSCx retrosplenial cortex, OCx occipital cortex, PCx parietal cortex, FCx frontal cortex, Th thalamus, Str striatum

The expression level of the microglia-specific allograft inflammatory factor 1 (*Aif1*, gene encoding protein IBA1) was analyzed using RT-PCR. In the saline-treated group, hHSPB1 transgenic animals had more than two times higher *Aif1* mRNA level (261%) compared to wild-types after 24 h (Fig. 3a). Ethanol administration increased the *Aif1* mRNA levels in wild-type animals (205%) 1 day after treatment, while no further increase was observed in the ethanol-treated transgenic pups (Fig. 3a). Morphological changes of microglial cells were studied using IBA1 immunostaining which was evaluated by deep learning methods. The highest proportions of resting microglial cells with highly ramified, fine processes were detected in the brain of the untreated animals; however, there were some differences observed between the wild-type and transgenic samples (Fig. 3b, c). Control transgenic animals had significantly smaller amounts of activated (intermediate and amoeboid) and higher amounts of resting microglia in the thalamus, and similar, albeit nonsignificant, changes were observed in the other brain regions as well (Fig. 3c; see Table S3 for the statistical analysis of the proportion of activated microglia). In contrast to saline treatment, 24 h after ethanol administration, we detected a high proportion of activated, hypertrophied microglia with increased cell bodies and shorter, thicker processes in the striatum as well as in the parietal and frontal parts of the cortex, whereas amoeboid microglia were only present in a smaller percentage in these areas. Contrarily, in the retrosplenial and occipital cortical regions or in the thalamus and in the hippocampus, we found a particularly high proportion of amoeboid microglia cells along with the intermediate ones (Fig. 3b, c). The proportion of intermediate and amoeboid microglia was significantly higher in all brain areas of the ethanol-treated mice compared to those of the control animals; however, the transgenic group showed a nonsignificant trend of increased proportion of activated microglia compared to ethanol-treated wild-type one (Fig. 3c). In parallel, the IBA1 coverage was more extensive in both ethanol-treated transgenic and wild-type brains compared to control ones. This increase in extent after ethanol treatment was more pronounced in the transgenic animals with significant differences in the frontal cortical, thalamic, and striatal regions (Fig. 3d). To determine the characteristic features of the activated microglia cells, we analyzed the expression of M1 (pro-inflammatory) and M2 (anti-inflammatory) phenotypic markers. In the case of M1 markers, only *Cd68* showed notable changes; as in response to ethanol treatment, a significant threefold increase (312%) was detected in hHSPB1 transgenic animals, whereas only a slight increase was observed in wild-type animals. Interestingly, the level of the other M1 marker, the inducible nitric oxide synthase (*iNos*), remained unchanged after treatment (Fig. 3e). Similar changes were observed for M2 markers. One of the markers, the mannose receptor C-type 1 (*Mrc1*), also remained unchanged, while the other one, arginase 1 (*Arg1*), was nearly doubled in wild-type animals (186%) and showed an even significantly higher, almost fourfold increase (375%) in hHSPB1 transgenic animals after ethanol treatment (Fig. 3e). One week after the treatment, microglial cells returned to their resting state-like morphology (Fig. S3a). Although *Aif1* mRNA levels were approximately the same in each group, 1 week after treatment, the IBA1-covered area remained smaller in most brain areas of ethanol-treated wild-type animals compared to those of the control wild-type group (Fig. S2d and Fig. S3b).

### Ethanol-induced activation of astrocytes in the brain of hHSPB1-overexpressing mice

**Fig. 4:**
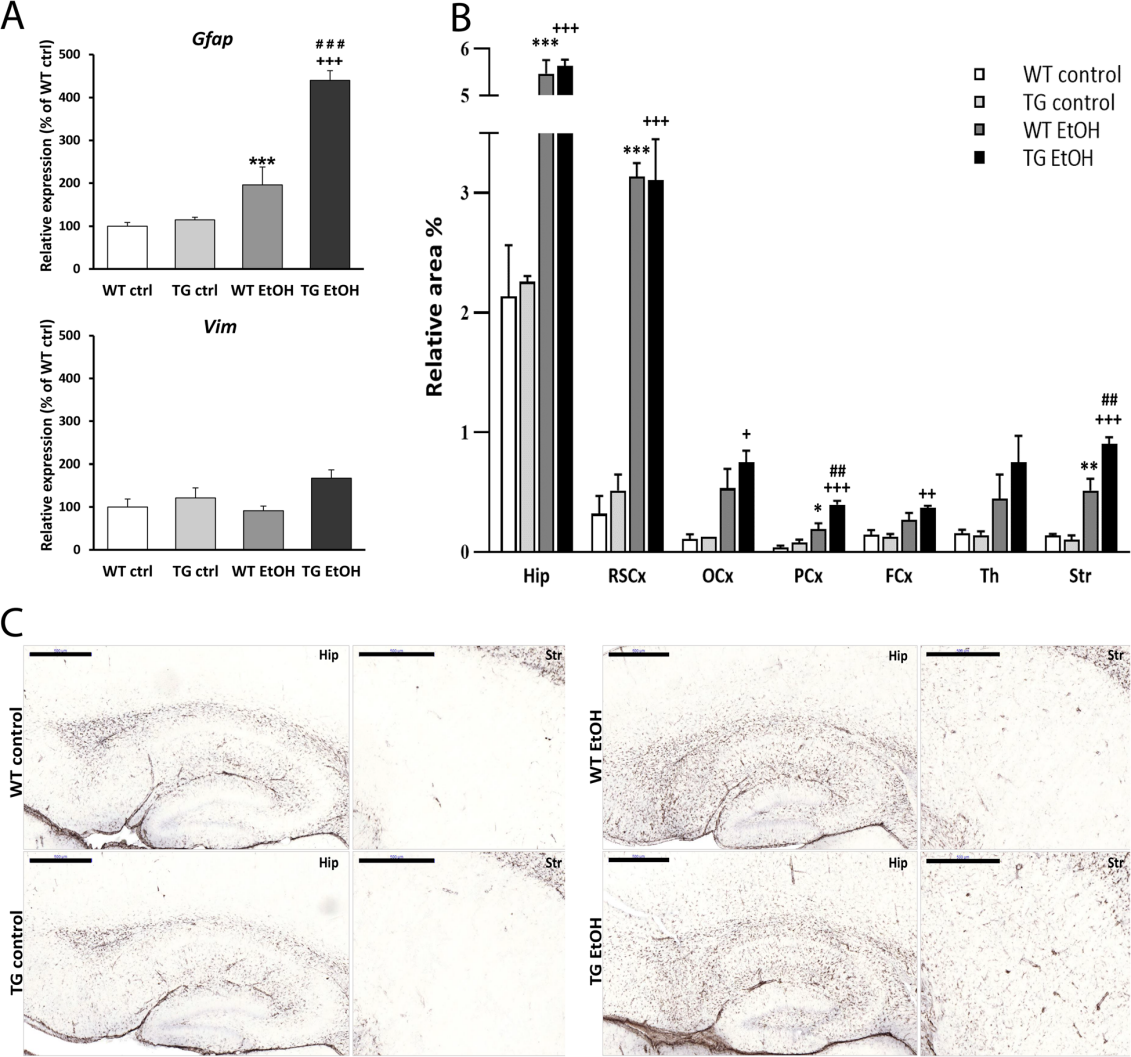
Astrocyte activation after ethanol treatment in the brain of transgenic and wild-type mice. **a** Relative expression of *Gfap* and *Vim* in the brain of hHSPB1-overexpressing and wild-type mice 24 h after ethanol treatment. Relative expression was correlated with the wild-type control group (100%). *n* = 8 mice per group. **b** Astrocytes with different morphologies were visualized with GFAP immunostaining using the peroxidase method 1 week after EtOH treatment. Quantification of GFAP immunoreactive areas: results are given in percentage of the immunopositive areas compared to the outlined brain areas (relative area). *n* = 3 mice per group, 3 sections per animal. **c** Morphological changes of astrocytes 1 week after EtOH treatment on sagittal brain sections of hHSPB1-overexpressing and wild-type mice. Scale bar: 500 μm. Values are presented as mean ± SEM. Asterisk indicates wild-type EtOH group vs wild-type control group; plus sign indicates transgenic EtOH group vs transgenic control group; hashtag indicates transgenic EtOH group vs wild-type EtOH group. Statistical analysis: 2-way ANOVA followed by Tukey post hoc test. *^/+^*p* < 0.05; **^/##/++^*p* < 0.01; ***^/###/+++^*p* < 0.001. Hip hippocampus, RSCx retrosplenial cortex, OCx occipital cortex, PCx parietal cortex, FCx frontal cortex, Th thalamus, Str striatum

In the next step, we studied the mRNA expression levels of *Gfap* and *Vim*, two intermediate filament proteins of astrocytes used as markers of reactive astrogliosis. The level of *Gfap* mRNA was increased 24 h after ethanol treatment both in the wild-type and transgenic groups, showing a fourfold increase in the transgenic group (440%) and a twofold increase in the wild-type one (196%, Fig. 4a). In contrast, the expression level of *Vim* mRNA did not change in the wild-type group and only slightly increased in the transgenic group in response to ethanol treatment (Fig. 4a). The level of both markers returned to the level of the control group 1 week after the treatment (Fig. S2e-f). The expression pattern of GFAP at the protein level and morphological changes of astrocytes was studied using immunohistochemistry. No significant change was detected in the morphology of astroglia 24 h after ethanol treatment (Fig. S4a-b). One week later, however, an increased GFAP immunoreactivity and hypertrophic astrocytes were detected in different brain regions both in wild-type and transgenic mice in response to ethanol treatment (Fig. 4b, c). Quantification of the immunostained area revealed a strong enhancement in GFAP coverage throughout the brain 1 week after ethanol treatment. A significant difference between ethanol-treated wild-type and transgenic mice was found in the striatum and in the parietal cortex (Fig. 4b).

### Changes in the level of ethanol-induced apoptosis in HSPB1-overexpressing transgenic mice

**Fig. 5:**
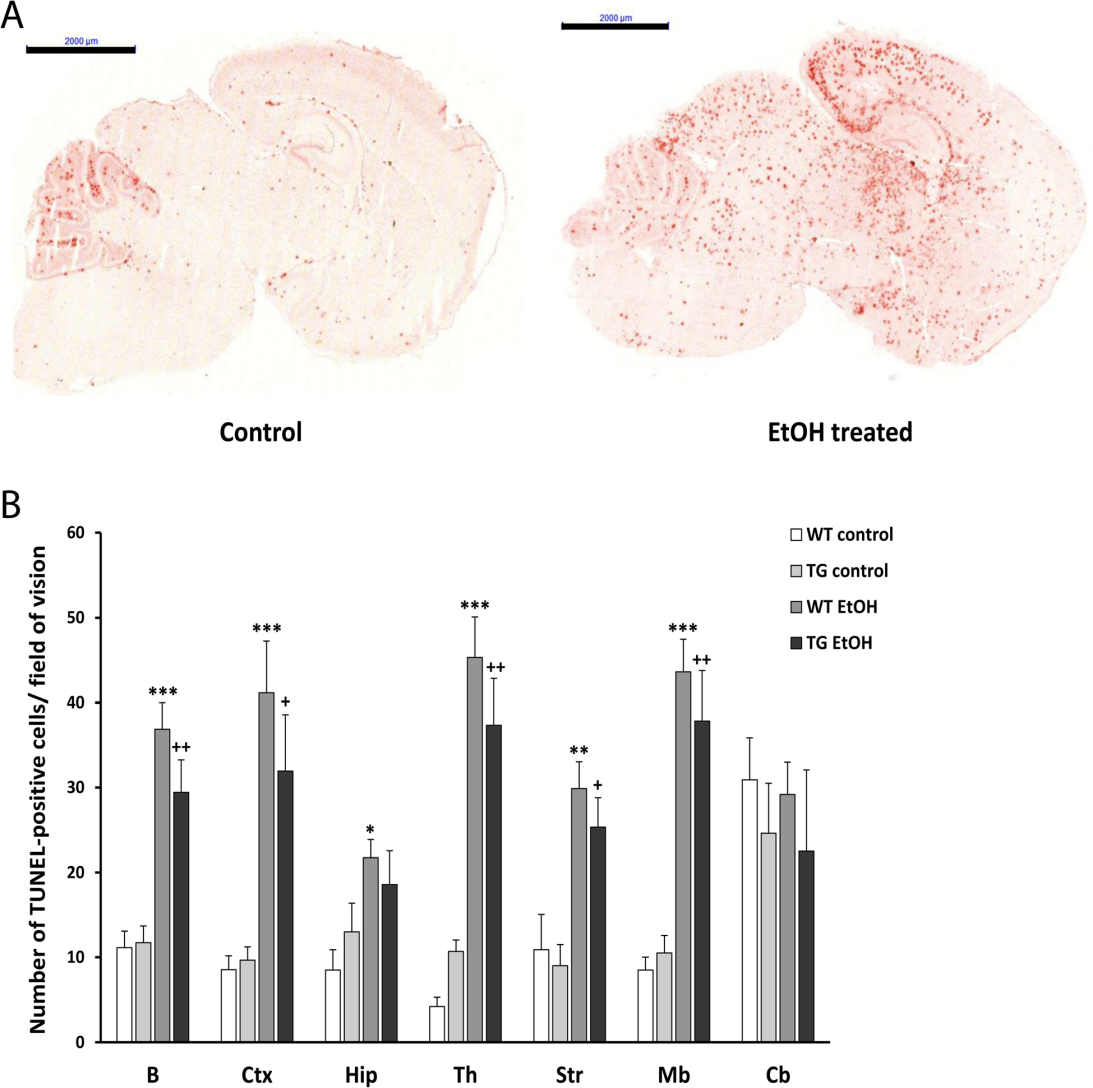
Apoptotic cell death detection using TUNEL assay 24 h after EtOH treatment. **a** Programmed cell death was detected on sagittal brain sections of control and EtOH-treated hHSPB1-overexpressing and wild-type mice after 24 h of the respective treatment. Scale bar: 2000 μm. **b** Apoptotic cells were counted in the brain of transgenic and wild-type mice 24 h after EtOH treatment. Values are presented as mean ± SEM; *n* = 6 mice per group. Asterisk indicates wild-type EtOH group vs wild-type control group; plus sign indicates transgenic EtOH group vs transgenic control group. Statistical analysis: 2-way ANOVA followed by Tukey post hoc test. *^/+^*p* < 0.05; **^/++^*p* < 0.01; ***p < 0.001. B whole brain, Ctx cortex, Hip hippocampus, Th thalamus, Str striatum, Mb midbrain, Cb cerebellum

In order to detect programmed cell death in the brain sections of mice, we analyzed the amount of fragmented DNA using TUNEL assay. In the brain of the control, saline-treated animals, a very low level of apoptosis was observed, while the number of TUNEL-positive cells was dramatically increased 24 h after ethanol treatment (Fig. 5a). For quantitative comparison, the TUNEL-positive cells were counted in fifteen areas of each brain section of each group. The statistical analysis revealed a significant increase in the number of apoptotic cells in most of the studied brain regions following ethanol treatment. The highest number of apoptotic cells was found in the cortex, thalamus, striatum, and midbrain, whereas a lower level of apoptosis was detected in the hippocampus. In the cerebellum, a high level of physiological cell death was visible, but the number of apoptotic cells remained similar after ethanol treatment (Fig. 5b). Comparing wild-type and hHSPB1-overexpressing animals, a slightly lower number of apoptotic cells was found in the brain of the transgenic animals; however, the difference was not statistically significant (Fig. 5b).

These in vivo results (summarized in Fig. S9) served as a basis for our subsequent in vitro experiments where we aimed to characterize the specific cell type that could be responsible for the aforementioned alteration of immune regulation triggered by the overexpression of hHSPB1.

### Characterization of primary neuronal, astrocyte, and microglial cell cultures derived from hHSPB1 and wild-type mice

**Fig. 6:**
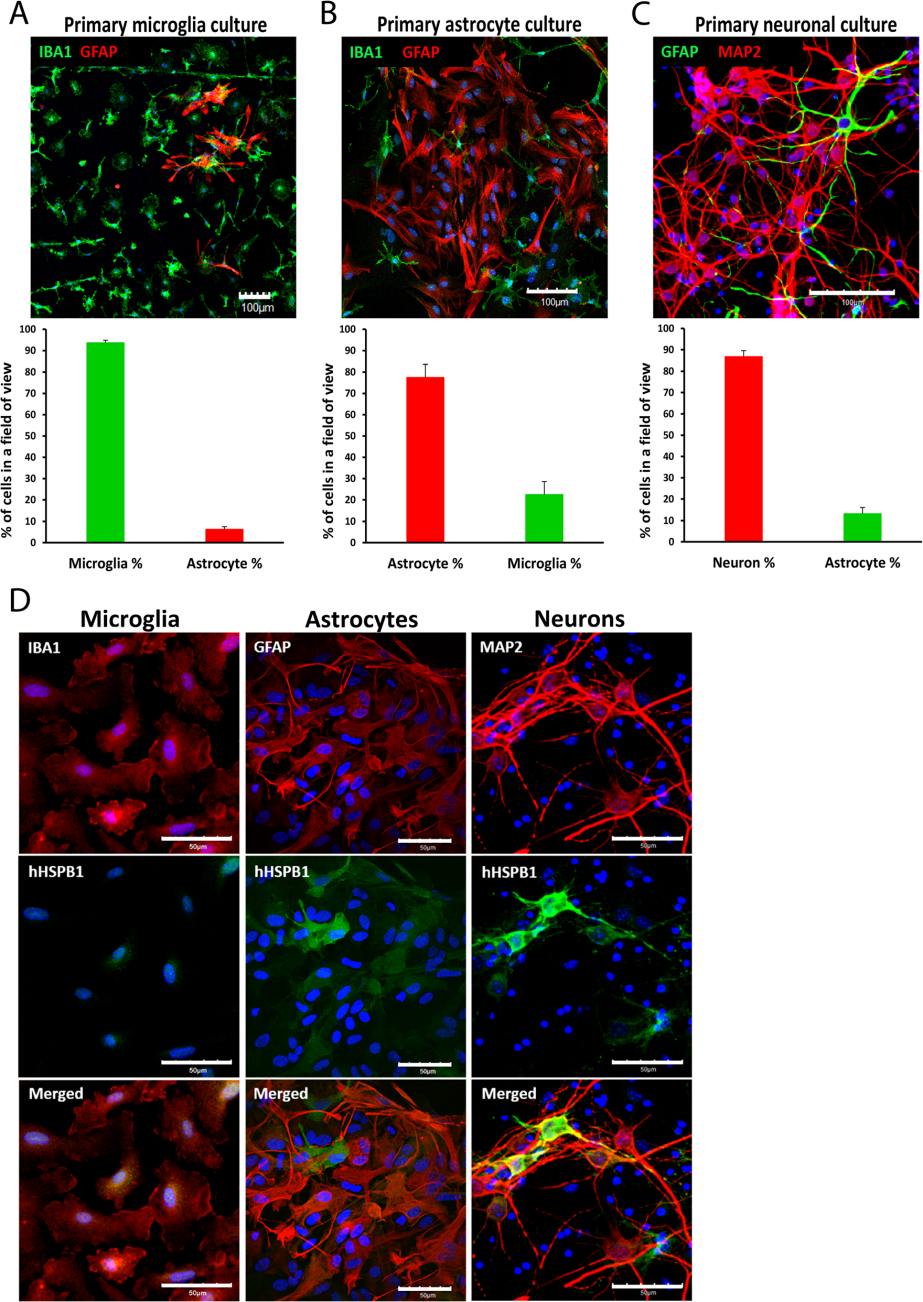
Characterization of the established primary cell cultures. Evaluation of the purity of primary **a** microglia, **b** astrocyte, and **c** neuronal cultures using fluorescent immunohistochemistry and detection of the presence of microglial (IBA1—green), astrocytic (GFAP—green/red), and neuronal (MAP2—red) markers. Cell nuclei were counted for cell-specific markers and percentages compared to the total cell number are given. Scale bar: 100 μm. Values are presented as mean ± SEM; *n* = 3–11. **d** Analysis of the expression pattern of the transgenic hHSPB1 protein in primary cell cultures using double immunofluorescent staining. Scale bar: 50 μm. Green: hHSPB1; red: IBA1/MAP2/GFAP; blue: DAPI

Primary neuron, astrocyte, and microglia cultures were prepared from the brain of hHSPB1 transgenic animals and from their wild-type littermates. First, the purity of the primary cultures was assessed by fluorescent immunohistochemistry, examining the presence of microglial (IBA1), astrocyte (GFAP), and neuronal (MAP2) markers. IBA1-GFAP double staining of the primary microglia culture confirmed that its purity exceeded 93% (Fig. 6a). We used the same double staining to determine the purity of the primary astrocyte culture, which indicated that the microglia cells were present at the cell culture at a proportion of 22% (Fig. 6b). The primary neuronal culture was labeled with MAP2 and GFAP antibodies, which showed that more than 85% of the cells were neurons in the culture (Fig. 6c). We also analyzed the expression pattern of the transgene in the primary cell cultures using hHSPB1-MAP2, hHSPB1-GFAP, and hHSPB1-IBA1 fluorescent immunostainings. The colocalization of the cell-specific markers with hHSPB1 was similar to what was observed in the brain sections earlier. We found that hHSPB1 colocalized with MAP2 and GFAP, but was rarely detectable together with IBA1, indicating that the transgenic hHSPB1 protein was mainly expressed by primary neurons and astrocytes (Fig. 6d).

### Effect of ethanol and cytokine treatment on the TNFα production of primary neuron, astrocyte, and microglia cultures

Primary cell cultures were treated with ethanol in order to investigate the effect of alcohol on different cell types of the brain. Ethanol can induce inflammatory processes in the brain directly by itself and also by its metabolites [33, 34], and it can also contribute to cerebral inflammation indirectly by disrupting the integrity of the blood-brain barrier, subsequently promoting the infiltration of peripheral immune cells to the brain [35]. Considering this affiliation of ethanol treatment with inflammation, we also treated our model with pro-inflammatory cytokines in order to model the inflammatory milieu connected with the presence of ethanol. The effective treatment concentrations of the cytokine and ethanol were selected based on the data available in the literature [21, 36] and on corresponding cell viability measurements. We used a concentration of 50 mM ethanol for astrocytes and microglia cells and 200 mM for neurons. During the cytokine treatment, the combination of 10 ng/ml human TNFα and 10 ng/ml human IL-1β was applied to all cell types (see Supplementary material and Fig. S5 for further details).

**Fig. 7:**
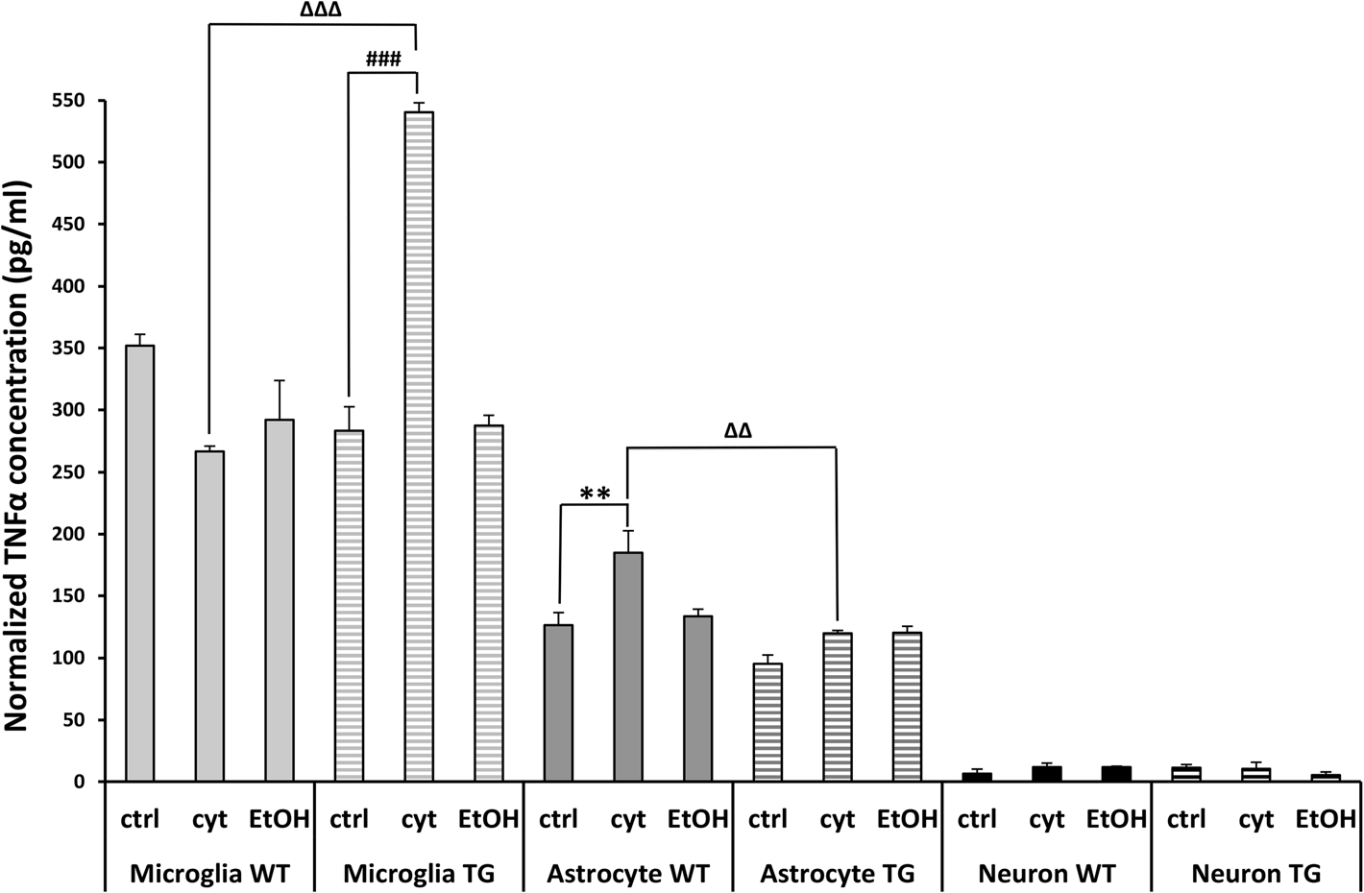
Quantification of TNFα production of primary cell cultures. Twenty-four hours after EtOH and cytokine treatment, cell culture supernates were collected. Concentrations of released TNFα (pg/ml) in the supernate of all the three isolated primary cell cultures were quantified using ELISA. TNFα concentrations were normalized to cell number of microglia and to RTCA values of astrocytes and neurons. crtl untreated control, EtOH ethanol treatment, cyt cytokine treatment. Values presented are means ± SEM; *n* = 2–3. Asterisk indicates wild-type cytokine vs wild-type control group; hashtag indicates transgenic cytokine vs transgenic control group; white triangle indicates transgenic cytokine vs wild-type cytokine group. Statistical analysis: 2-way ANOVA followed by Tukey post hoc test. **^/ΔΔ^*p* < 0.01; ^###/ΔΔΔ^*p* < 0.001

In order to analyze which cell type is the main source of TNFα and to confirm whether hHSPB1 regulates cytokine production in different brain-derived cells in our model, we performed an enzyme-linked immunosorbent assay (ELISA) assay specific against TNFα using the supernates from primary cultures. The TNFα concentration values were normalized to the corresponding cell viability measurements: to cell number for microglia and to RTCA values for astrocytes and neurons. Under control conditions, TNFα was detectable in the highest proportion in the supernates of glial cells. Microglial cells had almost three times higher basal expression of TNFα (TG group: 283 pg/ml; Wt group: 351 pg/ml; columns 1–6, Fig. 7) compared to astrocytes (TG group: 95 pg/ml; Wt group: 126 pg/ml; columns 7-12, Fig. 7), indicating that microglia are the primary source of TNFα in the brain. Cytokine treatment significantly increased the release of TNFα from hHSPB1-expressing transgenic microglial cells compared to nontreated transgenic cells (from 283 to 540 pg/ml). However, no such effect was observed in wild-type microglia. This indicates that the overexpression of hHSPB1 might modulate the release of TNFα since microglia from hHSPB1 transgenic animals expressed a significantly higher level of TNFα after cytokine treatment than the cytokine-treated wild-type group (540 pg/ml and 266 pg/ml, respectively; Fig. 7). Contrarily, after cytokine treatment, TNFα production was significantly higher in wild-type astrocytes compared to the control group; however, it remained unchanged in the hHSPB1 transgenic cells resulting in a significant difference between the two genotypes upon cytokine treatment (WT: 184 pg/ml and TG: 120 pg/ml, respectively; Fig. 7). Surprisingly, ethanol treatment only did not elicit a response from either glial cell type (Fig. 7). In the supernates of primary neurons, a very low TNFα presence was observed under control conditions (Fig. 7), and no change was seen in response to the various treatments.

### Effect of cytokine and ethanol treatments on extracellular and intracellular hHSPB1 levels

**Fig. 8:**
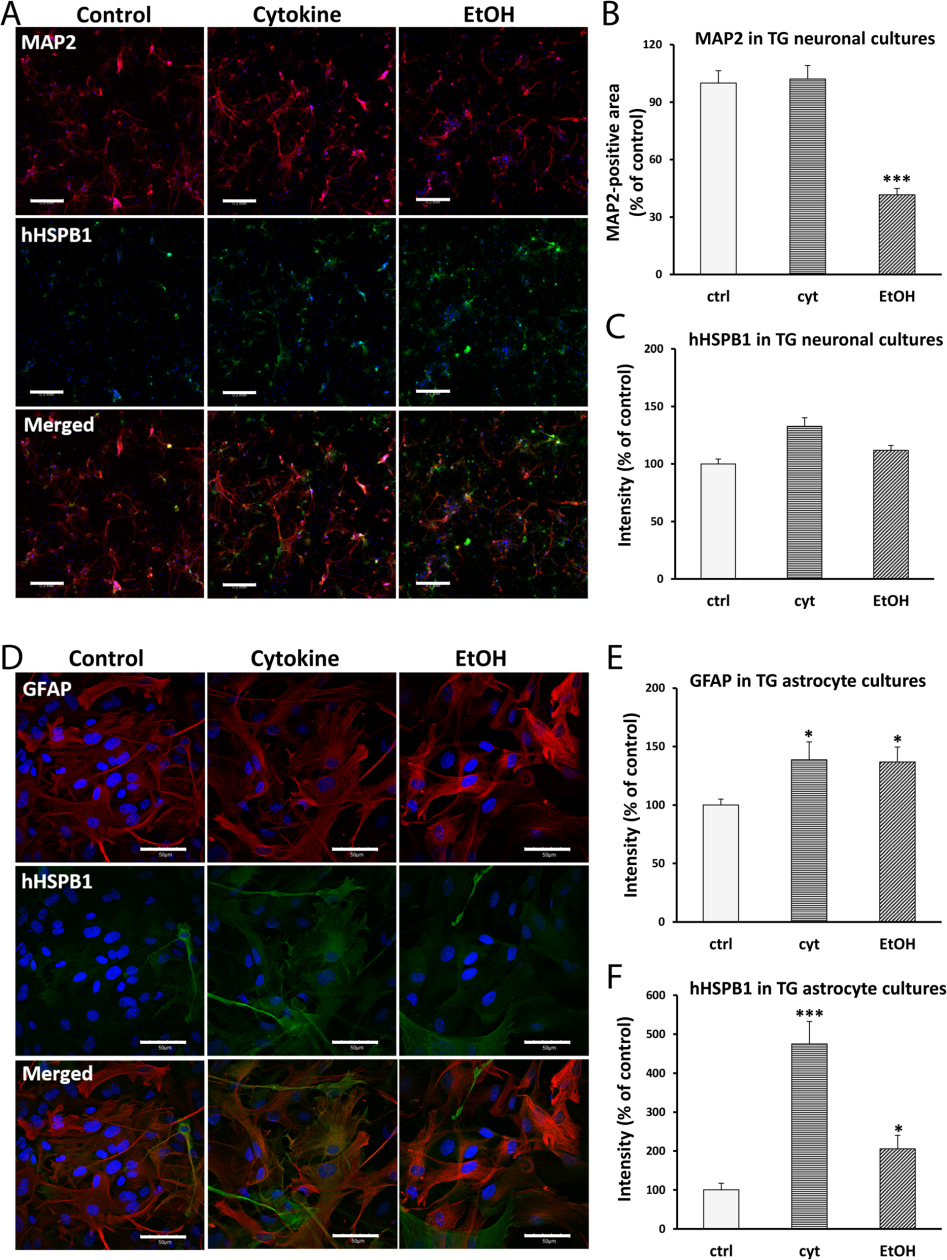
Effects of cytokine and EtOH treatment on intracellular hHSPB1, MAP2, and GFAP levels in transgenic cultures. **a** hHSPB1 and MAP2 double immunofluorescence staining in primary neuronal culture from hHSPB1-overexpressing mice 24 h after EtOH and cytokine treatment. Scale bar: 200 μm. Red: MAP2; green: hHSPB1; blue: DAPI. **b** Quantification of MAP2-positive area in transgenic primary neurons. *n* = 12. **c** Quantification of hHSPB1 fluorescent intensity in transgenic primary neurons. *n* = 12. **d** hHSPB1 and GFAP double immunofluorescence staining in primary astrocyte culture from hHSPB1-overexpressing mice 24 h after EtOH and cytokine treatment. Scale bar: 50 μm. Red: GFAP; green: hHSPB1; blue: DAPI. **e** Quantification of GFAP fluorescent intensity in transgenic primary astrocytes. *n* = 9–12. **f** Quantification of hHSPB1 fluorescent intensity in transgenic primary astrocytes. *n* = 10–11. Data are expressed as a percentage of untreated cells. Values presented are means ± SEM. crtl untreated control, EtOH ethanol treatment, cyt cytokine treatment. Statistical analysis: unpaired two-tailed *t* test. *p < 0.05; ***p < 0.001 compared to control

To determine whether extracellular or intracellular hHSPB1 elicits the proposed inflammation-regulating effects, we examined the level of hHSPB1 protein in the cell culture supernates by ELISA and in the cells by immunohistochemistry. The results of the human HSPB1 ELISA showed that no detectable amount of hHSPB1 was released by any of the analyzed cell types, upon treatment or under control conditions (Fig. S6). As microglial cells did not express an appreciable amount of intracellular hHSPB1 even after treatment (Fig. S7), we analyzed the changes in the intracellular level of the transgenic protein only in primary astrocytes and neurons. We found that the expression level of the transgene within primary neurons and astrocytes was influenced by the different treatments. In the case of primary neurons, the size of the MAP2-positive area was not altered by cytokine treatment, while it was significantly reduced by the ethanol treatment in both transgenic and wild-type neuronal cultures (to 41% and 46%, respectively) (Fig. 8a, b, Fig. S8a-b). In HSPB1 transgenic neurons, the fluorescence intensity corresponding to hHSPB1 remained unchanged after ethanol treatment, whereas it showed a moderate, but not significant increase after the cytokine treatment (Fig. 8a, c). In astrocyte cells, treatment with cytokines or ethanol elevated the intracellular GFAP levels. The cytokine treatment resulted in a significant increase in GFAP intensity in both transgenic and wild-type cells (to 138% and to 131%, respectively), whereas after the exposure to ethanol, a significant change was detected only in the transgenic astrocytes (136 %), but not in wild-type ones (Fig. 8d, e, Fig. S8c-d). In transgenic astrocytes, the level of hHSPB1 expression elevated significantly to 475% well above the control’s baseline expression after cytokine treatment and up to 205% after the ethanol treatment (Fig. 8d, f).

## Discussion

The neuroprotective role of HSPB1 in both acute and chronic neurodegenerative disorders has been previously demonstrated by multiple research groups including ours [20, 37, 38]. In parallel, a number of studies, using either mouse models of peripheral inflammation or cell cultures, revealed the regulatory function of HSPB1 in inflammation. In these studies, HSPB1 was affiliated with either pro- or anti-inflammatory functions in a context-dependent manner [2]. By expanding our knowledge on HSPB1 function as an immunoregulatory factor, it could become a potential therapeutic target for fine-tuning neuroinflammation, which is a common pathological characteristic of most neurological disorders. Therefore, in the present study, we investigated the immunoregulatory role of hHSPB1 in neuroinflammation using our previously established hHSPB1-overexpressing mouse model, as well as using primary cell cultures isolated from the brain of these transgenic animals.

The brain is extremely sensitive to the cytotoxic effect of ethanol during synaptogenesis, the period of brain development when it undergoes rapid growth. Neurons are vulnerable to ethanol-induced damage at this period to such an extent that even a single day of alcohol treatment can lead to neurodegeneration in the brain. Ikonomidou and colleagues [21] demonstrated that maintaining the blood ethanol concentration in 7-day-old rat pups above a toxic threshold (200 mg/dl) for at least 4 h triggers apoptotic cell death detectable 24 h after the treatment. Besides apoptosis, acute ethanol overdose has been demonstrated to trigger microglia and astrocyte activation in different animal models, so it is also suitable for investigating neuroinflammation [39]. Therefore, in this study, the same experimental arrangement was used to induce brain damage in wild-type and hHSPB1-overexpressing transgenic mice. In our transgenic animals, the human HSPB1 gene expression is driven by a cytomegalovirus (CMV) promoter. It is usually referred to as a constitutively active promoter; however, it has been shown to be upregulated in vitro under certain stress or inflammatory conditions [40–42]. Accordingly, increased transgene expression was observed in response to ethanol treatment in different brain regions of our transgenic mice and in the primary cells as well. Several previous studies have demonstrated that HSPB1 is upregulated upon neurological disorders; therefore, we used this transgenic strain to model the role of HSPB1 in neuroinflammation. Moreover, the expression pattern of hHSPB1 protein in this transgenic model is similar to the one that physiologically occurs in the human brain, as the hHSPB1 protein was found to be synthesized in astrocytes and neurons; however, hHSPB1-positive microglia were rarely detected [43].

Consistent with previous data, our results confirmed that even a single day of ethanol treatment is able to induce high level of apoptosis accompanied by glial cell activation and cytokine production in the developing brain. The presence of hHSPB1 in transgenic animals in itself did not influence the gene expression level of pro-inflammatory cytokines remarkably. However, hHSPB1 overexpression in combination with ethanol treatment resulted in a significantly higher increase in the level of pro-inflammatory cytokines compared to those observed in ethanol-treated wild-type mice 24 h after ethanol injection, whereas the level of anti-inflammatory cytokines remained unchanged. These results indicate that hHSPB1 overexpression intensifies the expression of pro-inflammatory cytokines in vivo following acute ethanol treatment.

Pro-inflammatory cytokines are produced in the brain by various cells, such as microglia. At the same time, cytokines affect microglia resulting in their further activation. Upon different immunological stimuli and neuronal injuries, microglia show an immediate response leading to altered morphology and function. These activated microglia cells are implicated in the protection of brain tissue by cleaning cellular debris via phagocytosis, and in inflammatory processes as well [39]. Immunostaining with IBA1 antibody clearly showed that the activation of microglia takes place within 24 h after a single day of ethanol treatment, as the size of IBA1-positive areas and the proportion of activated microglia cells increased significantly in all brain regions in response to ethanol. Ethanol-treated hHSPB1 mice showed significantly greater IBA1 coverage in certain brain areas than their wild-type littermates, with a nonsignificant trend towards higher proportion of activated microglia. Interestingly, we found differences in the control groups as well, since the thalamus of control transgenic animals showed a significantly higher amount of ramified microglia and lower IBA1 immunoreactivity compared to that of wild-type ones. Our immunohistochemistry data seems to contradict the result of the RT-PCR experiments, where we found a higher gene expression of *Aif1* in the control transgenic vs wild-type group, while it did not show a further increase in the transgenic animals in response to ethanol treatment. This discrepancy could be derived from the different samplings in the two experiments: in the immunostaining, we analyzed certain brain regions separately, while mRNA was isolated from the whole-brain homogenates for the RT-PCR study. Following brain injury, activated microglia move rapidly towards the damaged neurons leading to a remarkable increase in the local microglial density [44]. Therefore, it could be possible that *Aif1* expression is overall invariable in the brain while there are local differences in certain brain regions, similarly what we found in the brain of the transgenic animals at the protein level with and without ethanol treatment. The alterations in the nontreated group could be the result of the fact that HSPB1 is involved in neurodevelopment, contributing to neuronal differentiation and angiogenesis [45]. However, when examining the effect of ethanol treatment, we must acknowledge that activated microglia could play a complex and, in many cases, context-dependent role in the damaged nervous system, as they can contribute to both pathological and reparative processes. Recent studies showed that the depletion of microglia was able to block the expression of ethanol-induced pro-inflammatory factors [46, 47], whereas other groups found that the elimination of microglia exacerbated neuronal damage and augmented pro-inflammatory processes after ischemic brain injury [48–50]. In our study, although higher IBA1 immunoreactivity and higher pro-inflammatory cytokine expression were found in ethanol-treated transgenic animals compared to their wild-type littermates, there was no concomitant increase in neuronal loss, suggesting that hHSPB1 overexpression may induce diverse or even opposite microglial functions. Therefore, we examined the expression of markers corresponding to M1/M2 microglial polarization and we found that, in response to ethanol treatment, hHSPB1 overexpression enhanced the presence of both microglial phenotypes, since the M2 microglia marker *Arg1* and the M1 marker *Cd68* were also significantly higher in transgenic animals compared to wild-type ones. However, the remarkably high expression of pro-inflammatory cytokines demonstrated that M1 (pro-inflammatory) microglia predominated in hHSPB1 transgenic animals 1 day after ethanol treatment. After ethanol-induced brain injury, microglial recovery could be important in promoting neurogenesis leading to functional recovery [51, 52]. One week after the ethanol treatment, IBA1-stained microglia have recovered their highly ramified processes throughout the brain, accompanied by the decrease of cytokine expression to the normal level. It seems that in the ethanol-treated wild-type animals, microglial recovery could not take place within 1 week, while the level of IBA1 was almost equal in the transgenic groups with or without ethanol treatment.

Astrocytes, the other key cells in neuroinflammation, are special glial cells that have many essential functions in the brain. They respond to different forms of brain injuries through a process called reactive astrogliosis, which has different stages, according to the severity of the injury [53]. The morphological transformation of astrocytes and glial scar formation are partly controlled by pro- and anti-inflammatory cytokines [44]. As ethanol treatment triggered a rapid increase in cytokine expression and microglia activation in our experiment, it is not surprising that *Gfap* mRNA level was also elevated 24 h after treatment, showing significantly higher expression in ethanol-treated transgenic animals compared to ethanol-treated wild-type ones. However, no substantial changes were found neither in the morphology of the astrocytes nor in the GFAP coverage on the first day after injection. Instead, a remarkable expansion of GFAP-stained cells was observed 1 week later, corresponding with earlier studies showing that the formation of GFAP-positive filaments is a slower process. Moreover, GFAP staining around the blood vessels was also augmented in response to ethanol, especially in hHSPB1-overexpressing transgenic brains, leading to a significant difference in the percentage of GFAP-covered area between the two genotypes in certain brain regions. We also studied the inflammatory processes in primary astrocyte cultures in which astrocyte activation was observed, as we detected a pronounced GFAP upregulation within the cells in response to cytokine and ethanol treatments. Astrocytes can have a multitude of functions in the brain; thus, the remarkably high astrocytic hHSPB1 expression in the present study could potentially modulate these functions and could be key in understanding our observations. HSPB1 has been shown to interact with GFAP and to assist the proper organization of the filament network in astrocytes, which potentially contribute to the maintenance of normal astrocyte function [12]. Furthermore, strong colocalization of HSPB1 with cleaved caspase-3 in reactive astrocytes could promote astroglial survival [54]. HSPB1 may not only have positive effects in glial cells but can also be beneficial for the surrounding damaged tissue [55]. For example, Bechtold and Brown [56] raised the possibility of a synaptic transfer of HSPB1 from glial cells to neurons under stress conditions. In addition, presynaptic localization of HSPB1 in astrocytes could also promote neuronal survival via the restoration of synaptic activity after stress stimuli [56].

We also analyzed which cell type of the brain tissue could be responsible for the highly elevated levels of pro-inflammatory cytokines in hHSPB1 transgenic mice. Our in vitro results demonstrated that microglia are the primary TNFα-producing cell type under inflammatory conditions. In terms of astrocytes, both wild-type and transgenic cells had a basal TNFα production, which increased after cytokine treatment in the wild-type culture, but not in the transgenic one. Considering that HSPB1 was previously shown to interact with Annexin A1 and thereby lower the release of TNFα in reactive astrocytes [17, 57], it is possible that the greatly elevated level of intracellular hHSPB1 in astrocytes may directly affect their TNFα production. The observation that ethanol treatment did not induce TNFα release in glial cultures may be explained by the fact that the inflammation-inducing effect of ethanol is partly related to neuronal damage. For example, in the brain of adult rodents, which are less sensitive to the harmful effects of ethanol compared to fetuses, only a low degree of neurodegeneration or decline in neurogenesis was observed and, in parallel, no significant changes in protein levels of pro-inflammatory cytokines were detected [22, 58]. On the other hand, the effects of ethanol are potentiated by its metabolites: acetaldehyde had a greater impact on astrocyte and oligodendrocyte cultures than ethanol [59, 60]. Overall, these findings are in accordance with previous studies, in which activated microglia were identified as the main source of pro-inflammatory cytokines, whereas TNFα production by astrocytes was lower even after activating stimuli [61, 62]. We consider the use of serum for the culture of our primary cells as a limitation of our study because of potential priming effects. We should note that based on our present results as well as literature data, primary astro- and microglia cells despite being exposed to serum showed low expression of inflammatory mediators and activation markers [63, 64]. It is very interesting that, after cytokine treatment, microglia from hHSPB1 transgenic animals were seemingly in a further increased inflammatory status by secreting a higher amount of TNFα than wild-type cells, although microglia did not express appreciable amount of the transgene. This finding suggests a potential indirect effect of hHSPB1 on microglia activation. Based on previous data, not only the initial microglia response can affect the subsequent astrocyte activation, but microglial function could also depend on the crosstalk with astrocytes. For example, NFκB-mediated astrocytic expression of WNT induces microglia proliferation or the ATP released from astrocyte can act on the purinergic receptors of microglia with concomitant induction of Ca^2+^ signaling and IL-1β containing vesicula formation [65–68]. Astrocytes can also mediate microglial function and distribution by secreting a plethora of inflammatory cytokines and chemokines or through direct cell-cell interactions [69–71]. Therefore, we hypothesize that elevated intracellular hHSPB1 levels in astrocytes remaining in microglial cultures could have an effect on the release of these factors, contributing to the increased microglial activation. HSPB1 has been demonstrated to regulate several signaling cascades, such as inflammation-associated NFκB or p38 mitogen–activated protein kinase (MAPK) pathways; therefore, it could modulate the expression of numerous factors. Intracellular HSPB1 was also shown to induce the transcription of inflammatory mediators by participating in the stabilization of their mRNA [72]. In addition, astrocyte cell culture treated with HSPB1 exhibited an increased level of IL-8 and decreased production of TGF-β1 and CD40 ligand [16]. However, based on these findings, it would be of interest to fully elucidate the activation circuit between astrocytes and microglia in hHSPB1-overexpressing mice.

These results clearly demonstrate that the overexpression of hHSPB1 protein promotes glial activation and cytokine response upon acute ethanol treatment. Despite that HSPB1 was previously seen as an anti-inflammatory factor, our observation is in accordance with a growing number of evidence suggesting a more complicated, context-dependent role for this protein in inflammation [73]. An earlier report using HeLa cells showed that HSPB1 is associated with the expression of pro-inflammatory factors and involved in the IL-1 and TNF-induced signaling [72]. In C6 glioma cells, the chaperone activity of HSPB1 contributed to the production of the pro-inflammatory cytokine, IL-6, whereas T cells from HSPB1^−/−^ mice exhibited reduced secretion of TNF and IL-2 [74, 75]. Recombinant HSPB1 treatment also resulted in enhanced pro-inflammatory cytokine expression in vitro [18, 76, 77]. Although the majority of the literature describes extracellular heat-shock proteins as regulators of inflammation, we could not detect hHSPB1 in the supernates of the primary cell cultures in our experiments. Thus, it is reasonable to suspect that the intracellular form of the protein is the major source of inflammation modulation in this scenario.

In order to learn more about the overall outcome of the amplified pro-inflammatory cytokine production in hHSPB1-overexpressing mice, ethanol-induced cell death was studied using TUNEL assay. In the brain of the control, saline-treated animals, the low extent of apoptosis could correspond to the physiological, programmed cell death occurring in the developing brain [21]. However, even a single day of ethanol treatment dramatically increased the number of apoptotic cells in most of the studied brain areas. The observed differences between the brain regions were corresponding to their previously described susceptibility to alcohol-induced cell death and to their described sensitivity to alcohol in different developmental stages [21]. Intriguingly, despite the remarkably high level of *Tnf*, we found a slightly lower amount of TUNEL-positive apoptotic cells in the brains of hHSPB1 animals compared with those of wild-types after ethanol treatment. TNFα is a described inducer of apoptosis in the nervous system acting in a dose-dependent manner [78–80]. On the other hand, numerous in vivo and in vitro studies proved that HSPB1 contributes to the prevention of neuronal cell death [81–84]. Previously, we demonstrated the protective effects of hHSPB1 on neurons after both acute and chronic ethanol treatment in adult mice [37]. The neuroprotective effect of the enhanced intracellular hHSPB1 could partly be based on its ability to interact with neurofilaments and with the actin network, and by this supporting the structural integrity of neurons [85, 86]. HSPB1 can also affect the TNFα-induced cell death. HSPB1 prevented TNFα-mediated apoptosis by modulating cellular glutathione levels [87], by mediating the activity of NFκB [88, 89], or the p38/extracellular signal-regulated kinase (ERK) signaling pathways [90]. Furthermore, HSPB1 was shown to prevent caspase-mediated apoptosis, which can also be initiated by TNFα [91, 92]. Based on these observations, we assume that the elevated level of hHSPB1 could compensate the apoptosis-inducing effect of the highly increased *Tnf* in the hHSBP1-overexpressing mice. Moreover, it is worth mentioning that elevated levels of pro-inflammatory cytokines in the nervous system can also have beneficial effects. For example, depending on the target receptor it acts on, TNFα can be involved in demyelination as well as in neuronal regeneration, whereas neurotrophic factors were found to be upregulated after acute IL-1β administration [93, 94]. Furthermore, it has been recently discovered that chronic IL-1β and TNFα treatment activates an astrocyte phenotype that supports neuronal viability [95].

## Conclusions

Taken together, our work provides in vivo evidence that hHSPB1 overexpression is associated with enhanced pro-inflammatory response in a mouse model of ethanol-induced acute neuroinflammation. Notably, hHSPB1 overexpression modulated the inflammatory processes evoked by ethanol resulting in higher expression of pro-inflammatory cytokines and increased glial cell activation. The findings presented here could shed light on an unknown balancing role of hHSPB1 in inflammation regulation as the increased inflammation in the hHSPB1-overexpressing mice was not accompanied by increased neuronal damage. Thus, the data presented by this study could help to understand the potential regulatory role of hHSPB1 in neuroinflammation.

## Supplementary Information

**Additional file 1.** Supplementary materials

## Data Availability

The datasets used and/or analyzed during the current study are available from the last authors on reasonable request.

## Abbreviations

Aif1: Allograft inflammatory factor 1
Arg1: Arginase 1
BSA: Bovine serum albumin
CD40: Cluster of differentiation 40
Cd68: Cluster of differentiation 68
DAB: 3,3′-Diaminobenzidine
DAPI: 4′,6-Diamidino-2-phenylindole
ELISA: Enzyme-linked immunosorbent assay
ERK: Extracellular signal–regulated kinase
FITC: Fluorescein isothiocyanate
Gapdh: Glyceraldehyde 3-phosphate dehydrogenase
GFAP: Glial fibrillary acidic protein
HSP: Heat-shock protein
HSPB1: Heat-shock protein B1
IBA1: Ionized calcium–binding adaptor molecule 1
IL: Interleukin
IκBα: Inhibitor of nuclear factor kappa B-α
iNos: Inducible nitric oxide synthase
MAP 2: Microtubule-associated protein-2
MAPK: Mitogen-activated protein kinase
Mrc1: Mannose receptor C-type 1
NEUN: Neuronal nuclear antigen
NFκB: Nuclear factor kappa B
OCT compound: Optimal cutting temperature compound
PBS: Phosphate-buffered saline
PBST: Phosphate-buffered saline with Tween 20
PFA: Paraformaldehyde
RIPA: Radioimmunoprecipitation assay
SDS: Sodium dodecyl sulfate
TGF-β1: Transforming growth factor-β1
TNFα: Tumor necrosis factor-α
TUNEL: Terminal deoxynucleotidyl transferase dUTP nick end labeling
Vim: Vimentin
