# Supplementary material for: Neuroinflammatory processes are augmented in mice overexpressing human heat-shock protein B1 following ethanol-induced brain injury
Source: J Neuroinflammation. 2021 Jan 10;18:22. doi: 10.1186/s12974-020-02070-2 (PMC7798334; doi:10.1186/s12974-020-02070-2)
Supplement: Supplementary file 1 — Additional file 1. Supplementary materials [file 12974_2020_2070_MOESM1_ESM.docx]

**Additional file 1.**

**Supplementary material to:**

**Neuroinflammatory processes are augmented in mice overexpressing human heat-shock protein B1 following ethanol-induced brain injury**

Brigitta Dukay^1,4, §^, Fruzsina R. Walter^2^, Judit P. Vigh^2^, Beáta Barabási^2,5^, Petra Hajdu^1^, Tamás Balassa^1,6^, Ede Migh^1^, András Kincses^2^, Zsófia Hoyk^2^, Titanilla Szögi^3^, Emőke Borbély^3^, Bálint Csoboz^1,7^, Péter Horváth^1,8^, Lívia Fülöp^3^, Botond Penke^3^, László Vígh^1^, Mária A. Deli^2^, Miklós Sántha^1*^ and Melinda E. Tóth^1*§^

^1^ Institute of Biochemistry, Biological Research Centre, Szeged, Hungary (Temesvári krt. 62., H-6726 Szeged, Hungary)

^2^ Institute of Biophysics, Biological Research Centre, Szeged, Hungary

^3^ Department of Medical Chemistry, Faculty of Medicine, University of Szeged, Szeged, Hungary

^4^ Doctoral School in Biology and ^5^ Doctoral School in Theoretical Medicine, University of Szeged, Szeged, Hungary.

^6^ Doctoral School of Informatics, ELTE Eötvös Loránd University, Budapest, Hungary

^7^ Institute of Medical Biology, University of Tromsø, Tromsø, Norway

^8^ Institute for Molecular Medicine Finland (FIMM), University of Helsinki, Helsinki, Finland

*shared last authors

^§^Correspondance should be sent to BD (dukay.brigitta@brc.hu) or MET (toth.erzsebetmelinda@brc.hu)

**Contents:**

Page 2 **Supplementary methods**

Page 3 **Supplementary results**

Page 4-14 **Supplementary figures**

Page 15-17 **Supplementary tables**

**Supplementary methods**

**Viability test**

In order to assess the viability of neurons and astroglial cells after cytokine and ethanol treatments we performed an assay suitable for the detection of cell viability without the need of the addition of a labeling agent. ACEA’s real-time cell electronic sensing (RTCA-SP, E-plate) (ACEA Biosciences, CA, USA) method is a sensitive, impedance measurement based assay, which uses a 96-well gold electrode embedded plate [1,2]. The measured impedance corresponds to cell shape change, cell death, and detachment from the bottom of the plate. The RTCA-SP system measures the attachment and growth of cells automatically at every 10 minutes defining the *cell index*, which is a representation of the background-corrected form of the impedance of the cell layers attached to the surface of the electrodes. Neurons and glial cells were passaged to the 96-well microtiter plates which were coated with poly-l and poly-d-lysine. For the measurement of the background impedance 50 µl culture medium was added to each well, then 50 µl cell suspension was seeded at a density of 6 × 10^3^ cells/well for astroglia and 1.8 × 10^4^ cells/well for neurons. When cell growth reached a plateau phase, they were treated with 10 ng/mL recombinant human TNFα and IL-1β and with 10-800 mM ethanol and impedance changes were registered for 24 hours (n=2-13). The compound used to show maximum cell death was Triton X-100 detergent at 1% concentration. Impedance of cells was compared to control wells.

**Resazurin-assay**

Resazurin-assay was used to determine the effective dose of ethanol treatment on neurons. Primary neurons were plated on poly-d-lysine coated black 96 well plates (Greiner Bio-one, Germany) at the density of 2 × 10^4^ cells/well. Cells were cultured with Neurobsasal/B27 complete medium not containing phenol-red. Primary neurons were treated 5 days after the isolation for 24 hours with the following ethanol concentrations: 0; 100; 200; 400; 800 mM. All ethanol concentrations were tested in 8 replicates. After 24 hours, the treating medium was replaced with medium containing 10% resazurin and incubated for 4 hours at 37 ̊C. Resazurin sodium salt (Sigma-Aldrich Ltd, Budapest, Hungary) was used to prepare the reagent. The fluorescence intensity of the samples was measured at 565/580 nm using a fluorescent microplate reader.

**Microglia viability**

To examine the effect of treatments on microglia viability, DAPI-labeled nuclei were counted manually on immunostained samples in 10–23 images per group to get cell density data. (For the detailed protocol of the staining procedure see Methods section “Fluorescent immunostaining”.)

**Supplementary results**

**Viability of isolated primary neuronal, astrocyte and microglial cell cultures originating from hHSPB1 and wild-type mice after cytokine and ethanol treatment**

To determine the effective treatment concentrations of cytokines and ethanol, viability kinetics of primary cells after treatment was examined by impedance measurement, which provides real-time assessment of cellular viability and adherence. Only astrocytes and neurons were analyzed with this technique, due to the low yield of primary microglia isolations. In the case of cytokine treatment, we applied the previously tested concentrations of 10 ng/ml TNFα and IL-1β combination to model the inflammatory environment [1]. Previous *in vivo* data have shown that the ethanol treatment was effective from above the threshold of 50 mM [3]. The 24-hour viability test using the E-plate showed that the 50 mM ethanol concentration was effectively decreasing impedance in astrocytes (Fig. S5a). Neurons were more resistant, and only 400 mM concentration led to a notable decrease in impedance (Fig. S5b), while another test performed with resazurin showed a significant decrease in metabolic activity from 200 mM ethanol treatment (Fig. S5c). This suggests, that metabolic effects start already from a 200 mM ethanol treatment, but shape change leading to lower adherence and viability reflected by decreased impedance only occurs from 400 mM ethanol treatment. Therefore, in our experiments, we used the 50 mM ethanol treatment concentration for glial cultures and 200 mM for neurons. Primary astrocyte and neuronal cell cultures responded to the cytokine treatment with a decreased cell index showing that the combination of 10 ng/ml TNFα and 10 ng/ml IL-1β caused a significant decline in their viability (Fig. S5a-b). In contrast to neurons and astrocytes, no significant cell death was found in primary microglia, however, transgenic cells showed a nonsignificant trend of decreasing cell number (Fig. S5d).

**Morphological and gene expression changes in primary astrocytes and microglia after cytokine and ethanol treatment**

During the immunostaining experiments, in parallel to the evaluation of the staining intensities for hHSPB1 transgene expression or for GFAP expression in astroglia, we also investigated the corresponding morphological changes of these cells. For both wild-type and transgenic groups, morphological changes occurred after treatments: cells developed more and thinner processes and cell bodies decreased in size (Fig. 8d and Fig. S8c). Microglia cells were also stained for IBA1 and hHSPB1 and changes in cell shape and processes were studied. In the case of microglia, we did not observe a change in IBA1 staining intensity. Here, after both cytokine and ethanol treatments, we found more processes, shrunken cell bodies and more perturbations were observed along the cell membrane (Fig. S7).

1 Harazin A, Bocsik A, Barna L, Kincses A, Váradi J, Fenyvesi F et al. Protection of cultured brain endothelial cells from cytokine-induced damage by α-melanocyte stimulating hormone. PeerJ 2018; 2018: e4774.

2 Barna L, Walter FR, Harazin A, Bocsik A, Kincses A, Tubak V et al. Simvastatin, edaravone and dexamethasone protect against kainate-induced brain endothelial cell damage. Fluids Barriers CNS 2020; 17. doi:10.1186/s12987-019-0166-1.

3 Ikonomidou C, Bittigau P, Koch C, Genz K, Stefovska V, Hörster F. Ethanol-induced apoptotic neurodegeneration and fetal alcohol syndrome. Science (80- ) 2000; 287: 1056–1060.

**Supplementary figures**

**
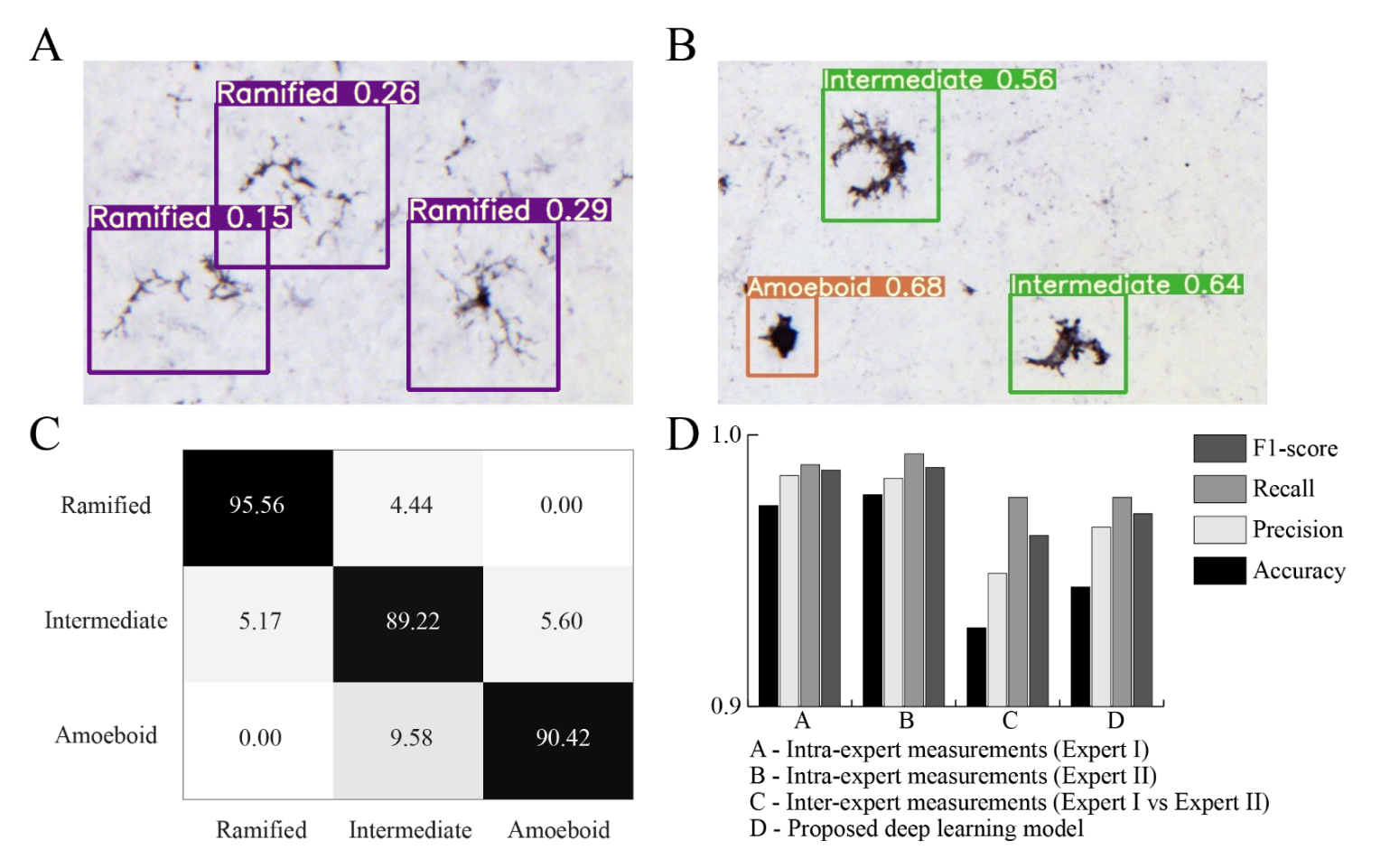
**

**Fig. S1 Evaluation of the performance of the deep learning method. a-b)** Representative images of cell detection and classification with FindMyCells. **c)** Confusion matrix of the classification results for the proposed method. **d)** Detection metrics for the human experts and the proposed method.

**
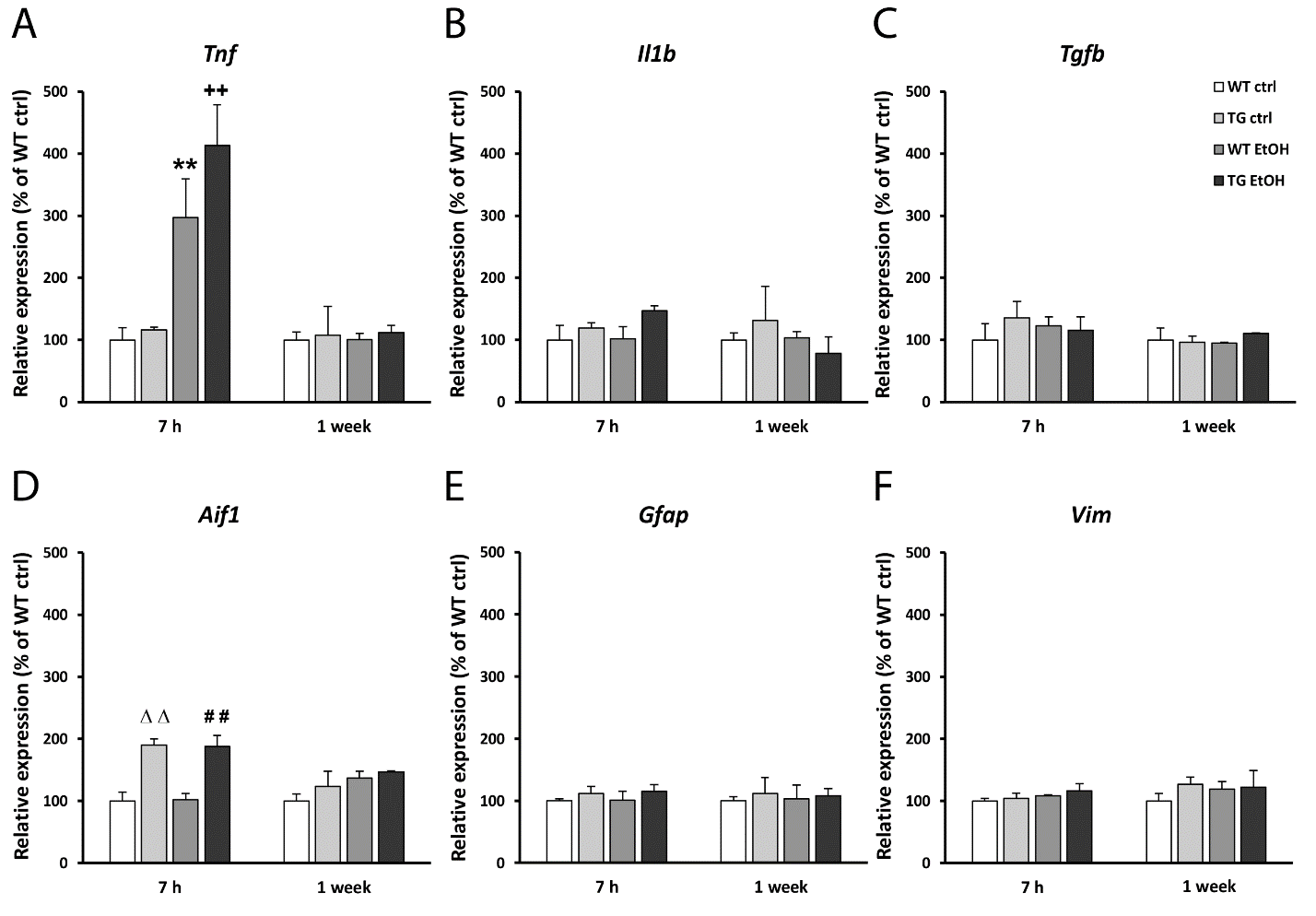
**

**Fig. S2. Gene expression analysis of inflammatory cytokines and glia activation markers in the brain**. Relative expression of **a)** *Tnf*, **b)** *Il1b*, **c)** *Tgfb*, **d)** *Aif1*, **e)** *Gfap*, and **f)** *Vim* was studied in the brain 7 hours or 1 week after the ethanol treatment using RT-PCR. Relative expression was correlated with the wild-type control group (100%). Data are represented as mean±SEM; n=3 mice per group. * indicates wild-type EtOH vs wild-type control group; + indicates transgenic EtOH vs transgenic control group; # indicates transgenic EtOH vs wild-type EtOH group, Δ indicates transgenic control vs wild-type control group. Statistical analysis: 2-way ANOVA followed by Tukey post-hoc test. **/##/++/ΔΔ denote p<0.01.

**
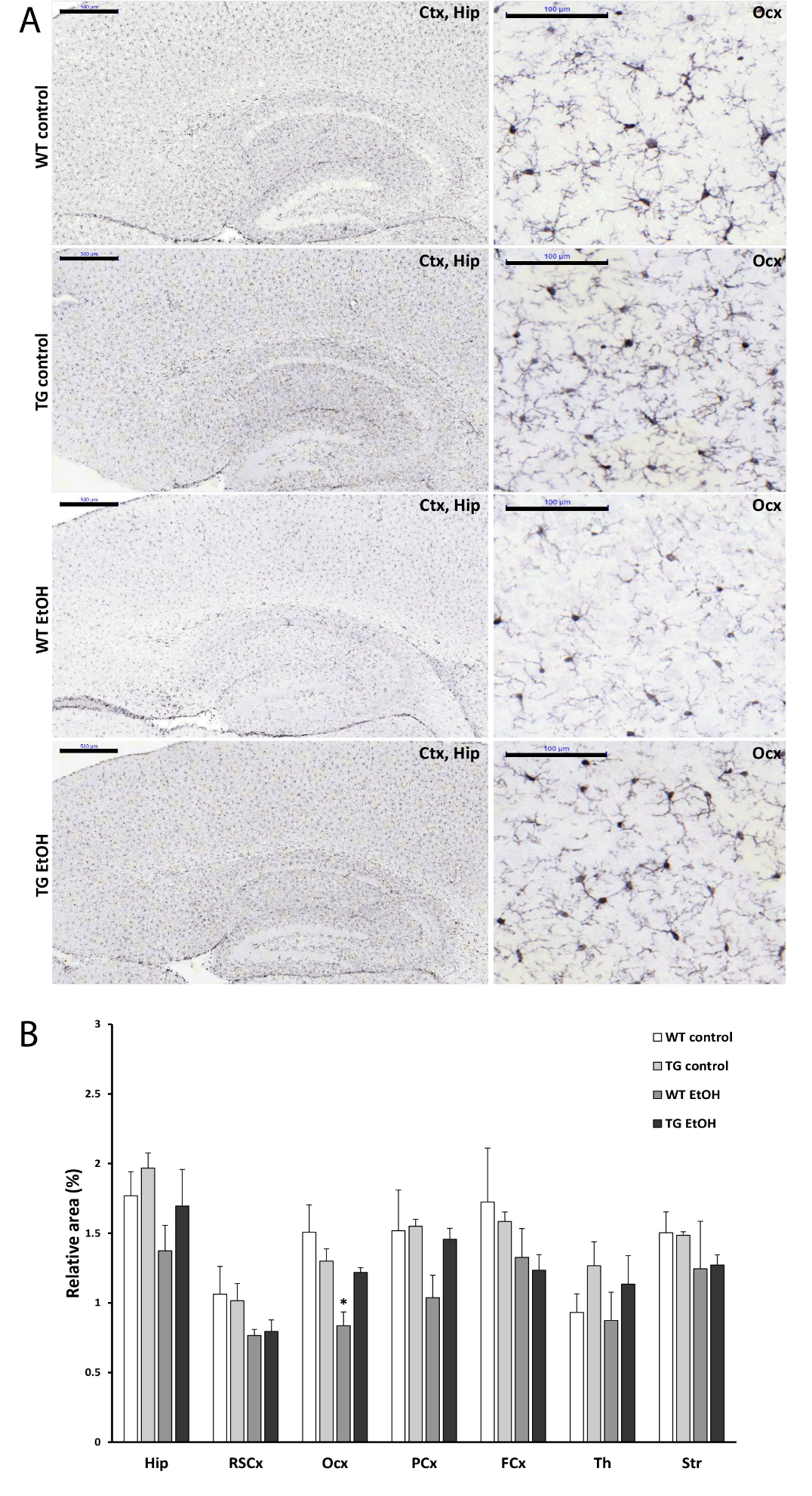
**

**Fig. S3. Microglia activation 1 week after ethanol treatment in the brain of transgenic and wild-type mice. a)** Morphological changes of microglia on sagittal brain sections of hHSPB1-overexpressing and wild-type mice 1 week after EtOH treatment. Microglia cells with different morphology were visualized with IBA1 immunolabeling using the peroxidase method. Scale bar: 500μm (Ctx, Hip) and 100μm (OCx). **b)** Quantification of IBA1 immunoreactive areas. Results are given in percentage of the immunopositive areas compared to the outlined brain areas (relative area). Data are represented as mean±SEM; n=3 mice per group, 3 sections per animal. Statistical analysis: 2-way ANOVA followed by Tukey post-hoc test. * indicates wild-type EtOH group vs wild-type control group. * denotes p<0.05. Abbreviations: *Ctx*: cortex, *Hip*: hippocampus, *RSCx:* retrosplenial cortex, *OCx*: occipital cortex*, PCx:* parietal cortex, *FCx:* frontal cortex, *Th:* thalamus, *Str*: striatum.


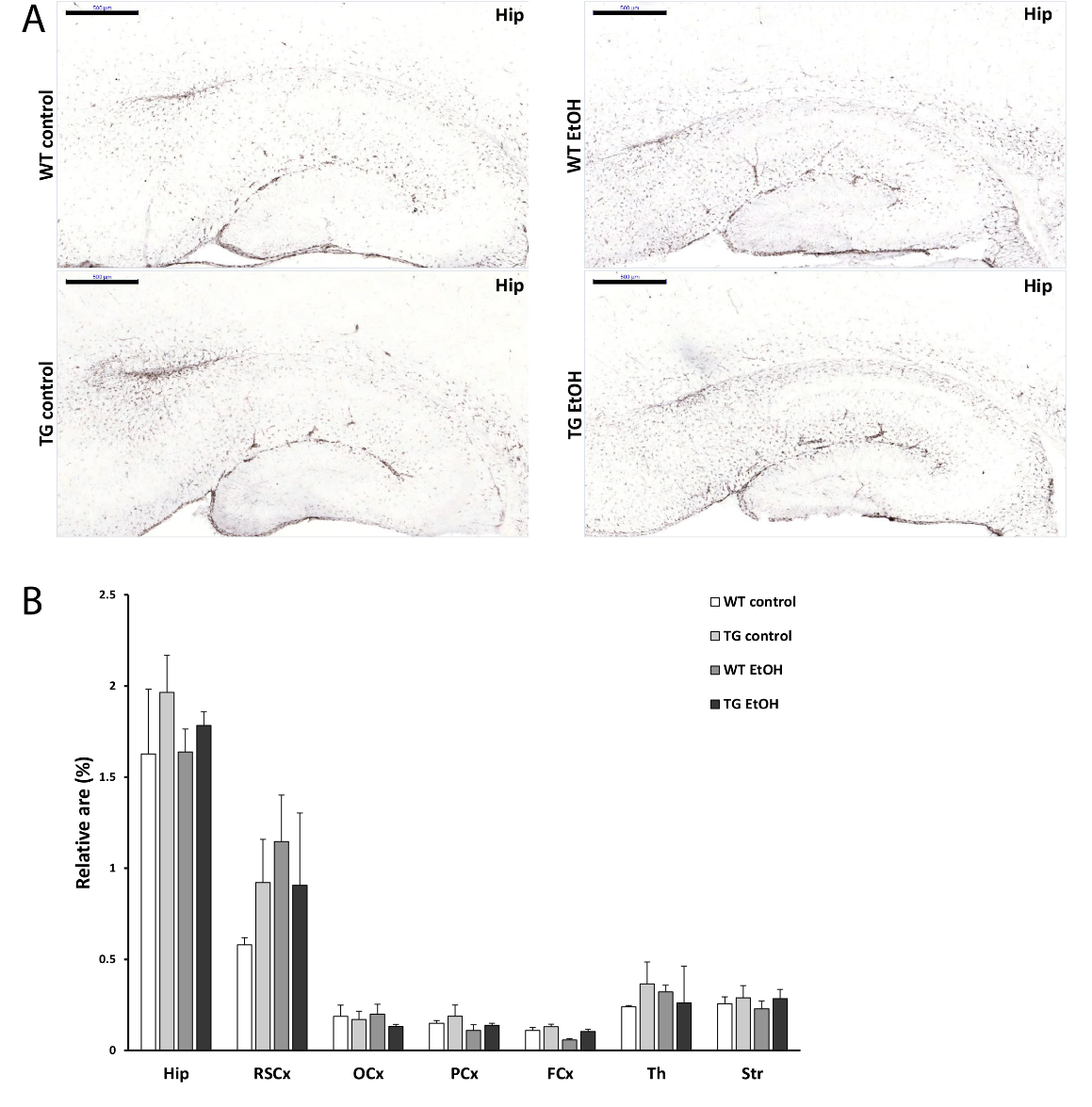


**Fig. S4. Astrocyte activation 24 hours after ethanol treatment in the brain of transgenic and wild-type mice. a)** Morphological changes of astrocytes 24 hours after EtOH treatment on sagittal brain sections of hHSPB1-overexpressing and wild-type mice. Astrocytes were visualized with GFAP immunostaining using the peroxidase method. Scale bar: 500μm. **b)** Quantification of GFAP immunoreactive areas. Results are given in percentage of the immunopositive areas compared to the outlined brain areas (relative area). Data are represented as mean±SEM; n=3 mice per group, 3 sections per animal. Abbreviations: *Hip*: hippocampus, *RSCx*: retrosplenial cortex, *OCx*: occipital cortex, *PCx*: parietal cortex, *FCx*: frontal cortex, *Th:* thalamus, *Str:* striatum.


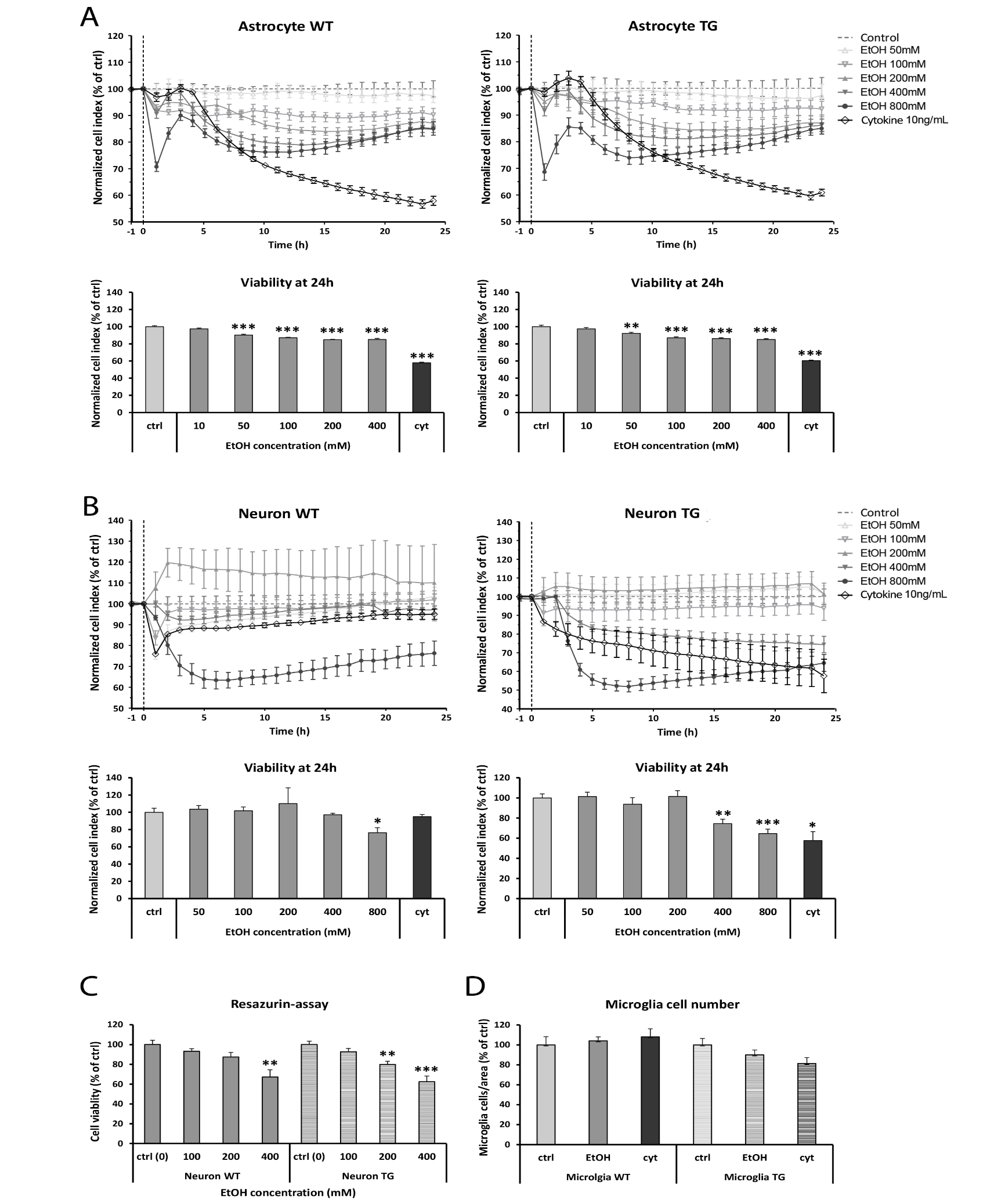


**Fig. S5. Viability of the primary cell cultures after ethanol and cytokine treatments**. Impedance measurement curves and results at 24 hours after EtOH and cytokine treatments in **a)** wild-type/transgenic astrocytes and in **b)** wild-type/transgenic neurons. **c)** Evaluation of the viability of primary neurons using Resazurin-assay 24 hours after EtOH treatment. **d)** Changes of microglia cell number 24 hours after EtOH treatment. Data are expressed as a percentage of untreated cells (100%). Values presented are means±SEM. Impedance measurement n=2-13; Resazurin-assay n=8. Quantification of cell number n=10-23. *crtl* untreated control, *EtOH* ethanol treatment, *cyt* cytokine treatment. Statistical analysis: unpaired two-tailed t-test. * denotes p<0.05; ** denote p<0.01; *** denote p<0.001 compared to control.


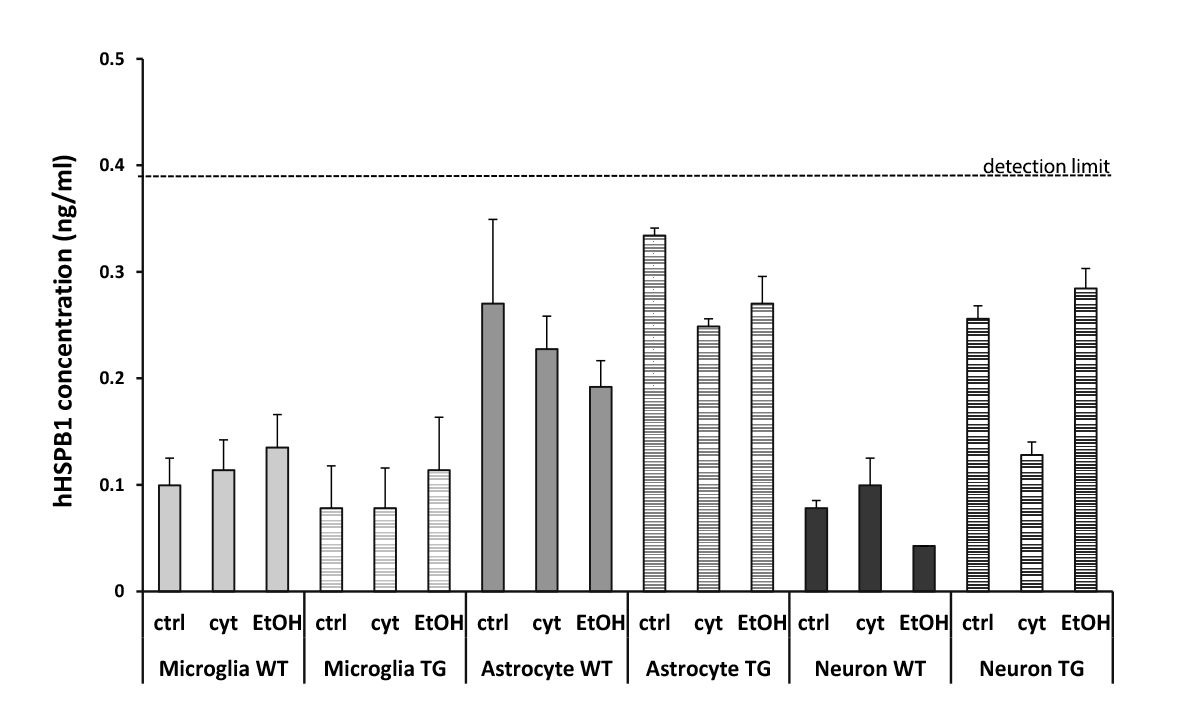


**Fig. S6. Quantification of hHSPB1 production of primary cell cultures.** Concentrations of released hHSPB1 (ng/ml) in the supernate of all three primary cell cultures were quantified using ELISA. *crtl* untreated control, *EtOH* ethanol treatment, *cyt* cytokine treatment. Values presented are means ± SEM; n=2-3. The dashed line indicates the lower limit of the sensitivity range of the hHSPB1 ELISA kit.

**
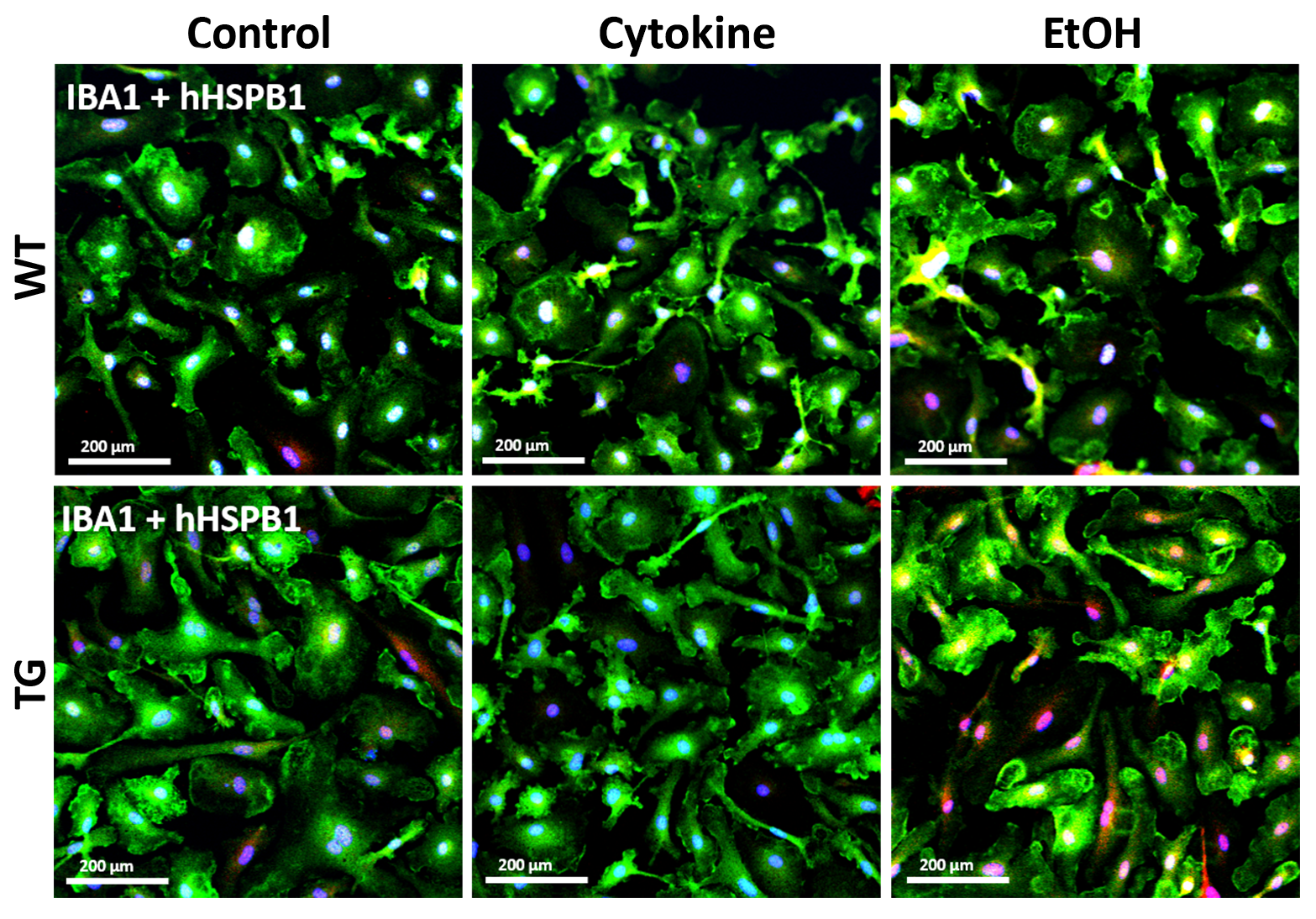
**

**Fig. S7. Transgenic hHSPB1 protein expression in primary microglia culture**. hHSPB1 and IBA1 double immunofluorescence staining in wild-type and transgenic microglia cells 24 hours after EtOH and cytokine treatment. Scale bar: 200μm. Red: hHSPB1. Green: IBA1. Blue: DAPI.

**
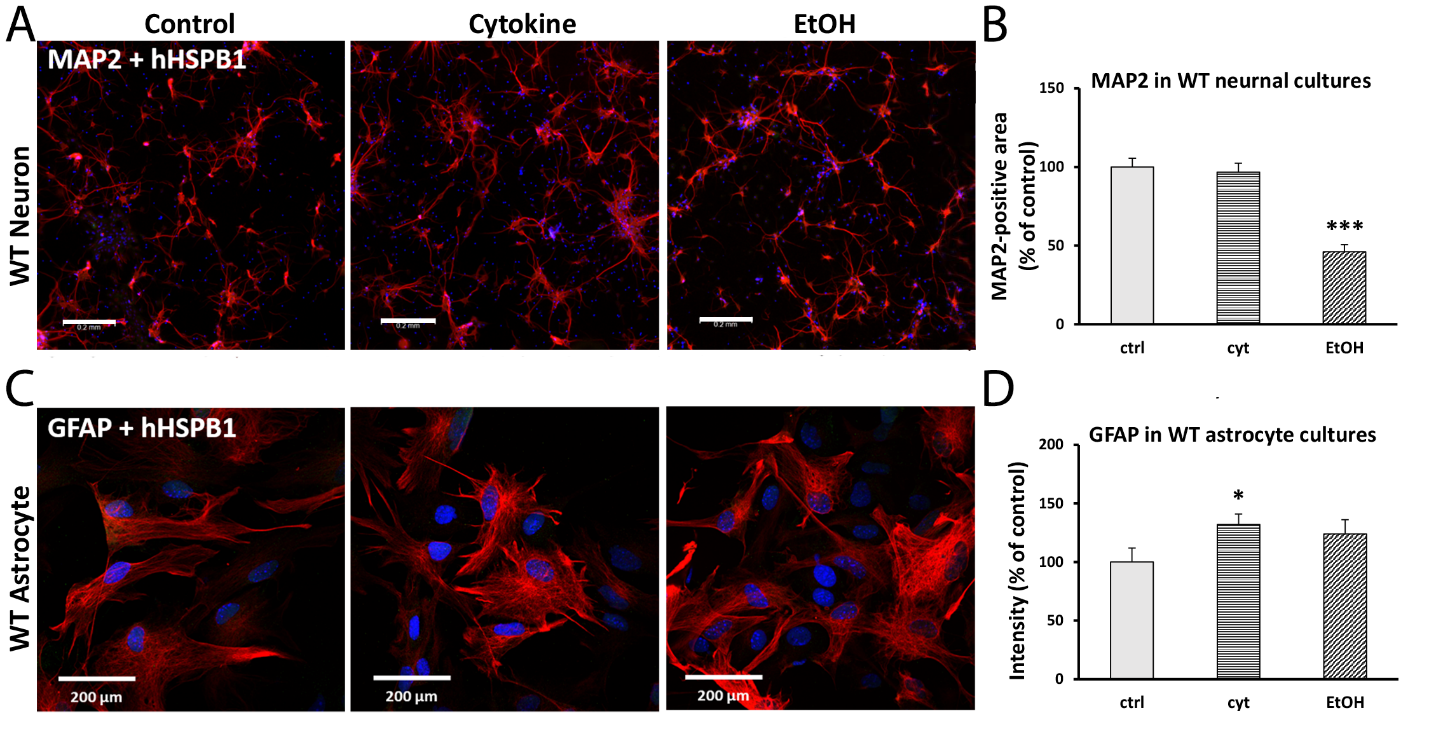
**

**Fig. S8. Transgenic hHSPB1 protein expression in wild-type neurons and astrocytes**. **a)** hHSPB1 and MAP2 double immunofluorescence staining in wild-type neurons 24 hours after EtOH and cytokine treatments. Scale bar: 200μm. Red: MAP2/GFAP. Green: hHSPB1. Blue: DAPI. **b)** Quantification of MAP2-positive area in wild-type primary neurons. n= 12. **c)** hHSPB1 and GFAP double immunofluorescence staining in wild-type astrocytes 24 hours after EtOH and cytokine treatments. Scale bar: 200μm. Red: MAP2/GFAP. Green: hHSPB1. Blue: DAPI. **d)** Quantification of GFAP fluorescent intensity in wild-type primary astrocytes. n=14-15. Data are expressed as a percentage of untreated cells. Values presented are means±SEM. Statistical analysis: unpaired two-tailed t-test. * denotes p<0.05; *** denote p<0.001 compared to control.


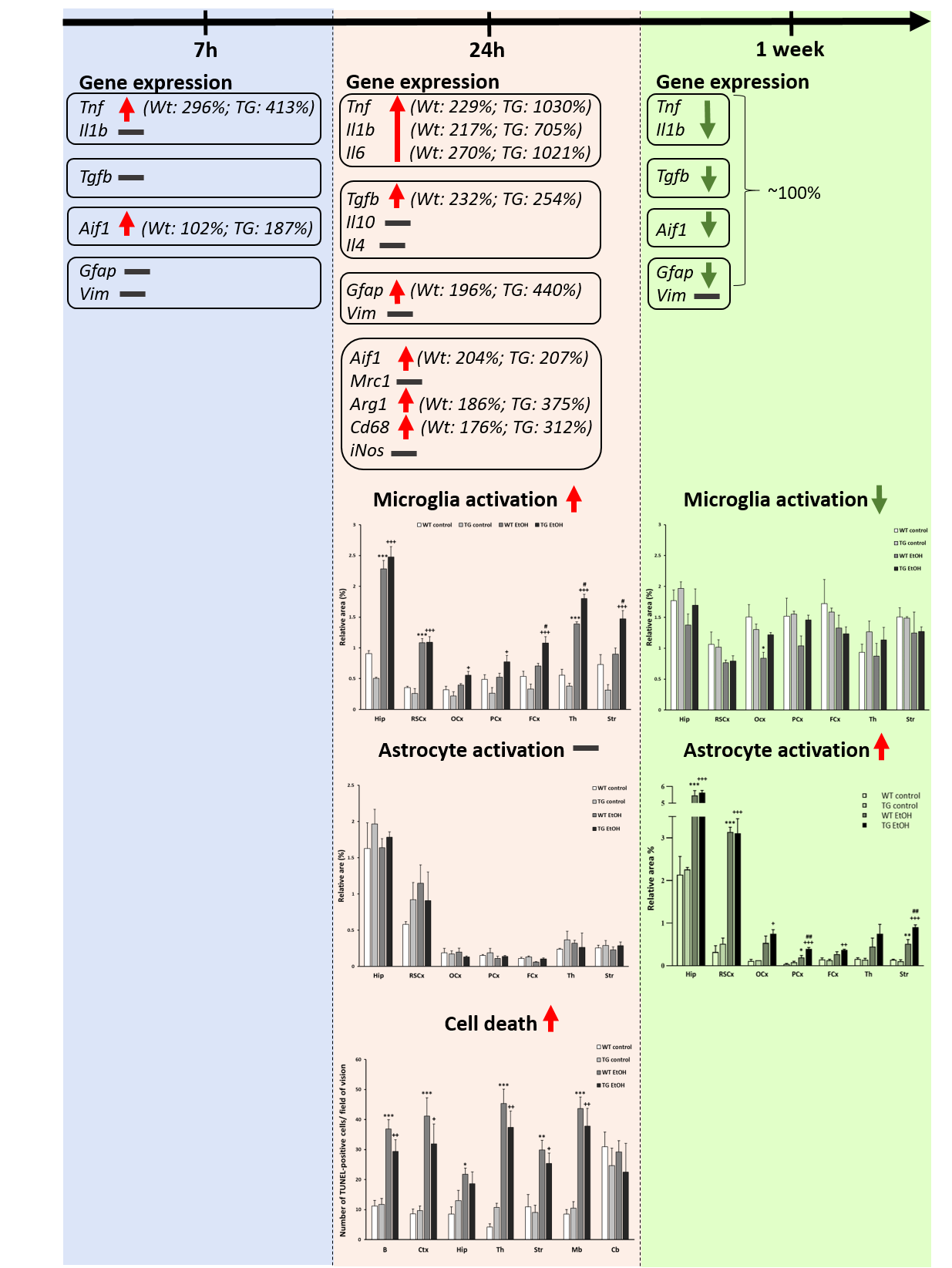


**Fig. S9. Summary of the ethanol-induced inflammatory processes in wild-type and hHSPB1-overexpressing transgenic mice**

In our early postnatal ethanol exposure model, the inflammatory processes started after 7h upon ethanol treatment, as the expression of *Tnf* showed a significant increase, the level of which was higher in hHSPB-overexpressing mice than in their wild-type littermates. The largest inflammatory response was detected at 24h after ethanol treatment, when an intense inflammation and cell death could be observed. The expression levels of pro-inflammatory cytokines, certain microglia, and astrocyte markers were also significantly higher at this time point in the transgenic group compared to the wild-type one. Moreover, hHSPB1-overexpressing animals showed significantly greater microglial coverage in the frontal cortex (FCx), thalamus (Th), and striatum (Str). However, these enhanced inflammatory responses in the transgenic mice were not accompanied by increased cell death. One week after ethanol treatment, the resolution of inflammation began, as the level of pro-inflammatory cytokines, and the expression of microglia and astrocyte markers decreased to their original level in both genotypes. Microglia cells also returned to their resting state-like morphology. However, ethanol-induced morphological changes in astrocytes were detectable at this time point and, similarly to microglia, we detected higher astrocyte coverage in the parietal cortex (PCx) and in the striatum (Str) of the hHSPB1 transgenic animals compared to their wild-type littermates.

**Supplementary tables**

**Table S1.** List of antibodies used in this study FITC: fluorescein isothiocyanate, A: Alexa Fluor

| **Antibody name** | **Vendor** | **Catalogue number** | **Host organism** | **Concentrations** | **Antibody registry ID** |
| --- | --- | --- | --- | --- | --- |
| **Western Blot analysis** | | | | | |
| anti-hHSPB1 | Stressgen | SPA-803 | rabbit | 2 µg/ml | [AB_2120611](http://antibodyregistry.org/AB_2120611) |
| anti-actin | Sigma | A2103 | rabbit | 0.25 µg/ml | [AB_476694](http://antibodyregistry.org/AB_476694) |
| horseradish peroxidase- labeled anti-rabbit | Jackson ImmunoResearch | 111-035-003 | goat | 0.02 µg/ml | [AB_2313567](http://antibodyregistry.org/AB_2313567) |
| **Fluorescent immunohistochemistry - Brain sections** | | | | | |
| anti-IBA1 | Abcam | ab5076 | goat | 0.5 µg/ml | [AB_2224402](http://antibodyregistry.org/AB_2224402) |
| anti-GFAP | Sigma | G3893 | mouse | 36.5 µg/ml | [AB_477010](http://antibodyregistry.org/AB_477010) |
| anti-NEUN | Millipore | MAB377 | mouse | 20 µg/ml | [AB_2298772](http://antibodyregistry.org/AB_2298772) |
| anti-hHSPB1 | Enzo Life Sciences | ADI-SPA-803 | rabbit | 20 µg/ml | [AB_10615084](http://antibodyregistry.org/AB_10615084) |
| A488 anti-goat | Jackson ImmunoResearch | 305-545-003 | rabbit | 5 µg/ml | [AB_2339532](http://antibodyregistry.org/AB_2339532) |
| FITC-anti-mouse | Sigma | F5387 | goat | 3.7 µg/ml | [AB_259647](http://antibodyregistry.org/AB_259647) |
| A647 anti-rabbit | Thermo Fisher Scientific | A21244 | goat | 6.7 µg/ml | [AB_2535812](http://antibodyregistry.org/AB_2535812) |
| **Peroxidase immunohistochemistry** | | | | | |
| anti-hHSPB1 | Enzo Life Sciences | ADI-SPA-803 | rabbit | 1.6 µg/ml | [AB_10615084](http://antibodyregistry.org/AB_10615084) |
| anti-GFAP | Sigma | G3893 | mouse | 18.25 µg/ml | [AB_477010](http://antibodyregistry.org/AB_477010) |
| anti-IBA1 | Abcam | ab5076 | goat | 0.5 µg/ml | [AB_2224402](http://antibodyregistry.org/AB_2224402) |
| horseradish peroxidase- labeled anti-rabbit | Jackson ImmunoResearch | 111-035-003 | goat | 0.4 µg/ml | [AB_2313567](http://antibodyregistry.org/AB_2313567) |
| peroxidase-labeled anti-mouse | Chemicon | AP160P | rabbit | 1:2000 | [AB_92531](http://antibodyregistry.org/AB_92531) |
| biotinylated anti-goat | Jackson ImmunoResearch | 705-065-147 | donkey | 1.3 µg/ml | [AB_2340397](http://antibodyregistry.org/AB_2340397) |
| **Fluorescent immunostaining - Cell cultures (culture purity assessment)** | | | | | |
| anti-MAP2 | Abcam | ab92434 | chicken | 0.25 µg/ml | [AB_2138147](http://antibodyregistry.org/AB_2138147) |
| anti-GFAP | Sigma | G3893 | mouse | 12.17 µg/ml | [AB_477010](http://antibodyregistry.org/AB_477010) |
| anti-IBA1 | Abcam | ab5076 | goat | 0.5 µg/ml | [AB_2224402](http://antibodyregistry.org/AB_2224402) |
| A594 anti-chicken | Jackson ImmunoResearch | 703-585-155 | donkey | 5 µg/ml | [AB_2340377](http://antibodyregistry.org/AB_2340377) |
| A488 anti-mouse | Invitrogen, Life Technologies | A21202 | donkey | 6.7 µg/ml | [AB_141607](http://antibodyregistry.org/AB_141607) |
| A488 anti-goat | Invitrogen | A11055 | donkey | 2 µg/ml | [AB_2534102](http://antibodyregistry.org/AB_2534102) |
| Dylight 549 anti-mouse | Jackson ImmunoResearch | 115-505-003 | goat | 0.94 µg/ml | [-](http://antibodyregistry.org/AB_258792) |
| **Fluorescent immunostaining - Cell cultures** | | | | | |
| anti-MAP2 | Abcam | ab92434 | chicken | 0.25 µg/ml | [AB_2138147](http://antibodyregistry.org/AB_2138147) |
| anti-hHSPB1 | Enzo Life Sciences | ADI-SPA-803 | rabbit | 2.5 µg/ml | [AB_10615084](http://antibodyregistry.org/AB_10615084) |
| anti-IBA-1 | Abcam | ab5076 | goat | 0.5 µg/ml | [AB_2224402](http://antibodyregistry.org/AB_2224402) |
| anti-GFAP | Sigma | G3893 | mouse | 3.65 µg/ml | [AB_477010](http://antibodyregistry.org/AB_477010) |
| A594-anti-chicken | Jackson ImmunoResearch | 703-585-155 | donkey | 5 µg/ml | [AB_2340377](http://antibodyregistry.org/AB_2340377) |
| A488-anti-rabbit | Thermo Fisher Scientific | A11034 | goat | 3.4 µg/ml | [AB_2576217](http://antibodyregistry.org/AB_2576217) |
| A488-anti-goat | Invitrogen | A11055 | donkey | 2 µg/ml | [AB_2534102](http://antibodyregistry.org/AB_2534102) |
| Cy3-anti-rabbit | Sigma | C2306 | sheep | 2.5 µg/ml | [AB_258792](http://antibodyregistry.org/AB_258792) |
| Dylight 488 anti-rabbit | Jackson ImmunoResearch | 111-485-003 | goat | 0.94 µg/ml | [-](http://antibodyregistry.org/AB_258792) |
| Dylight 549 anti-mouse | Jackson ImmunoResearch | 115-505-003 | goat | 0.94 µg/ml | [-](http://antibodyregistry.org/AB_258792) |

**Table S2.** Gene-specific primers for RT-PCR analysis

|  | **gene** | **forward primer** | **reverse primer** |
| --- | --- | --- | --- |
| **1** | ***Gapdh*** | GGG TTC CTA TAA ATA CGG ACT GC | CCA TTT TGT CTA CGG GAC GA |
| **2** | ***Tnfa*** | CCCTCACACTCAGATCATCTTCT | GCTACGACGTGGGCTACAG |
| **3** | ***Il1b*** | GCAACTGTTCCTGAACTCAACT | ATCTTTTGGGGTCCGTCAACT |
| **4** | ***Gfap*** | CGGAGACGCATCACCTCTG | AGGGAGTGGAGGAGTCATTCG |
| **5** | ***Aif1*** | ATCAACAAGCAATTCCTCGATGA | CAGCATTCGCTTCAAGGACATA |
| **6** | ***Vim*** | CGTCCACACGCACCTACAG | GGGGGATGAGGAATAGAGGCT |
| **7** | ***Tgfb*** | CTCCCGTGGCTTCTAGTGC | GCCTTAGTTTGGACAGGATCTG |
| **8** | ***hHSPB1*** | GTCCCTGGATGTCAACCACT | GACTGGGATGGTGATCTCGT |
| **9** | ***Il6*** | GCTACCAAACTGGATATAATCAGGA | CCAGGTAGCTATGGTACTCCAGAA |
| **10** | ***Il10*** | CAGAGCCACATGCTCCTAGA | TGTCCAGCTGGTCCTTTGTT |
| **11** | ***Il4*** | CATCGGCATTTTGAACGAG | CGAGCTCACTCTCTGTGGTG |
| **12** | ***Cd68*** | GACCTACATCAGAGCCCGAGT | CGCCATGAATGTCCACTG |
| **13** | ***iNos*** | GTTCTCAGCCCAACAATACAAGA | GTGGACGGGTCGATGTCAC |
| **14** | ***Mrc1*** | CCACAGCATTGAGGAGTTTG | ACAGCTCATCATTTGGCTCA |
| **15** | ***Arg1*** | GAATCTGCATGGGCAACC | GAATCCTGGTACATCTGGGAAC |

**Table S3.** Results of the morphological analysis of microglia 24 hours after ethanol treatment

(*p < 0.05, **p < 0.01, and ***p < 0.001, abbreviations: *Hip*: hippocampus, *RSCx*: retrosplenial cortex, *OCx*: occipital cortex, *PCx*: parietal cortex, *FCx*: frontal cortex, *Th:* thalamus, *Str:* striatum.)
